# Alkali‐Stable Metal–Organic Frameworks with Enhanced Electroconductivity for Black‐Brown Electrochromic Energy Storage Smart Window

**DOI:** 10.1002/advs.202407297

**Published:** 2024-10-01

**Authors:** Xinyi Wang, Zhiqiang Liu, Heqi Ma, Yiwen Liu, Qing Sui, Jifei Feng, Guofa Cai

**Affiliations:** ^1^ Key for Special Functional Materials of Ministry of Education, National & Local Joint Engineering Research Center for High‐Efficiency Display and Lighting Technology School of Nanoscience and Materials Engineering Henan University Kaifeng 475004 China

**Keywords:** conductivity, electrochromism, energy storage, metal–organic frameworks, smart window

## Abstract

Metal–organic frameworks (MOFs) deliver potential applications in electrochromism and energy storage. However, the poor intrinsic conductivity of MOFs in electrolytes seriously hampers the development of the above‐mentioned electrochemical applications, especially in one MOF electrode. Herein, a new Ni‐based MOF (denoted Ni‐DPNDI) is proposed with enhanced conductivity by π‐delocalized DPNDI connectors. Predictably, the obtained Ni‐DPNDI MOF achieves a conductivity of up to 4.63 S∙m^−1^ at 300 K. Profiting from its unique electronic structure, the Ni‐DPNDI MOF delivers excellent electrochromic and energy storage performance with a great optical modulation (60.8%), a fast switching speed (*t*_c_ = 7.9 s and *t*_b_ = 6.4 s), a moderate specific capacitance (25.3 mAh·g^−1^) and good cycle stability over 2000 times. Meanwhile, energy storage capacity is visual by the coloration states of Ni‐DPNDI film. As a proof of the potential application, a large‐area (100 cm^2^) electrochromic energy storage smart window is further designed and displayed. The strategy provides an interesting alternative to porous multifunctional materials for the new generation of electronic devices with diverse applications.

## Graphical abstract

A Ni‐based metal‐organic framework (named Ni‐DPNDI) with a conductivity of up to 4.63 S∙m^−1^ at room temperature is presented. Attributing to its unique electrical structure, the Ni‐DPNDI electrode delivers excellent electrochromic and energy storage performance, simultaneously. As a proof of practical application, a large area (100 cm^2^) of electrochromic energy storage window is further designed and constructed.

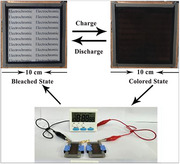

## 1 Introduction

Metal–organic frameworks (MOFs) present a kind of typical framework materials, which are constructed by the self‐assembly of metal nodes and organic connectors.^[^ ^1^, ^2^, ^3^, ^4^ ^]^ Taking advantage of their unique high porosity, tunable components, and high‐density active sites, MOFs are ideal electrode materials, especially in electrochromic (EC) and energy storage applications. Profiting from the above superiorities, MOFs can realize electron transport and ion diffusion in multiple dimensions to accelerate the electrochemical reaction.^[^ ^5^, ^6^, ^7^, ^8^, ^9^ ^]^ Unfortunately, the poor intrinsic electronic conductivity of MOFs still hampers their further applications in the electrochemical field.^[^ ^10^, ^11^ ^]^

To address the aforementioned issues, two main strategies are adopted currently. MOFs are employed as templates or precursors to obtain porous C, S, or N‐doped metal oxide materials, in which the graphene‐like frame can enhance the electron transport rate and stability.^[^ ^12^, ^13^, ^14^, ^15^ ^]^ However, the inherent structure of MOFs was destroyed in this way, resulting in pore structure collapse, active site aggregation, and the unavailability of the intrinsic superiority of MOFs. Subsequently, the construction of 2D MOFs with redox groups or delocalization π‐planar ligands enhances MOFs' conductivity.^[^ ^16^, ^17^ ^]^ Although 2D MOFs exhibit good electrochemical performance for EC and energy storage applications, the unsatisfactory stability limits their practical application.^[^ ^18^, ^19^ ^]^ Therefore, stable MOF materials with enhanced electroconductivity are essential for the development of MOFs for electrochemical application.

In recent research, MOFs have exhibited a preliminary application in electrochromism or energy storage. Since naphthalene diimide‐based EC MOFs material was developed by Dincă et. al., MOFs‐based EC materials have become an up‐and‐coming brilliance.^[^ ^20^ ^−^ ^24^ ^]^ For instance, Wang et. al designed a battery of EC MOFs materials derived from their constructed organic ligands of naphthalene diimide and perylene.^[^ ^22^, ^23^ ^]^ Fahar et. al. also constructed pyrene‐based EC material.^[^ ^24^ ^]^ Additionally, MOFs with redox‐active sites, such as Ni‐bpa,^[^ ^19^ ^]^Ni_3_(HITP)_2_,^[^ ^25^ ^]^ and Ni‐HAB,^[^ ^26^ ^]^ illustrate the potential in energy storage application. The above reports demonstrate electrochromic or energy storage applications in one MOF electrode. Nonetheless, the MOFs with electrochromism and energy storage simultaneously are neglected currently. Moreover, constructing MOFs with the above functions is important for their development in visual EC energy storage windows.

In this work, we present a new Ni‐based MOF (denoted Ni‐DPNDI) with a good conductivity of 4.63 S∙m^−1^ and robust chemical stability. In this structure, π‐delocalized DPNDI connectors are employed to increase the overlapped level of metal‐ligand orbital, enhancing the electroconductivity of MOFs. Benefiting from its excellent electrochemical performance, the Ni‐DPNDI delivers a dual‐functional characteristic of electrochromism and energy storage with great optical modulation (60.8%), fast response speed within 10 s, comparable specific capacitance (25.3 mAh·g^−1^), and good cycling stability over 2000 times. For highlighting practical applications of the Ni‐DPNDI MOFs, we further construct a large area (100 cm^2^) of EC energy storage smart window. The obtained smart window can realize the dynamic regulation of visible light and the reusable storage of electrical energy. Simultaneously, the state of charge can be monitored based on the coloration states of the smart window by the naked eye in real‐time. This work broadens the MOFs in application scenarios of visual energy storage, multifunctional smart windows, and other intelligent electronic devices.

## 2 Results and Discussion

**Figure 1:**
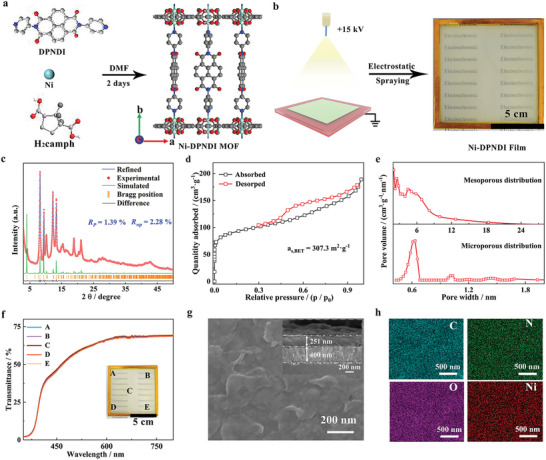
The synthetic process and structural characterization of Ni‐DPNDI MOF powder and the corresponding film. a) The scheme of preparation of Ni‐DPNDI MOF (Color code: gray, C; green, Ni; red, O; blue, N). b) The scheme of preparation of film by an electrostatic spray deposition. c) PXRD patterns of simulated and as‐prepared Ni‐DPNDI. N_2_ isotherms at 77 K d) and the pore diameter distribution e) of Ni‐DPNDI MOF. f) UV–vis transmittance spectra of Ni‐DPNDI film (100 cm^2^) at five different points of A‐E. g) Scanning electron microscopy (SEM) images of Ni‐DPNDI film. h) The distribution of Ni, O, C, and N elements during Ni‐DPNDI film.

The 2,7‐Di(Pyridin‐4‐Yl)Benzo[Lmn][3,8]Phenanthroline‐1,3,6,8(2H,7H)‐Tetraone (DPNDI) ligand was prepared via the amidation reaction of 4‐aminopyridine (Apy) and 1,4,5,8‐naphthalenetetracarboxylic dianhydride (NDI). The consistent nuclear magnetic resonance (^1^H NMR) and fourier transform infrared (FT‐IR) spectra with that in the literature verified the successful synthesis of the DPNDI ligand (Figures S1 and S2, Supporting Information).^[^ ^27^, ^28^ ^]^ Subsequently, the Ni‐DPNDI MOF was obtained by solvothermal preparation of Ni salt and the mixed ligands of DPNDI and DL‐(±)‐camphoric acid (H_2_camph) in *N*, *N*‐dimethylacetamide (DMF) according to the procedure (**Figure** **1**a, see Experiment Section in detail). The crystal structure of the Ni‐DPNDI MOF was demonstrated by theoretical structural simulation based on X‐ray diffraction (XRD) analysis (Figure 1c). The strong and sharp diffraction peaks of Ni‐DPNDI MOF agree well with the simulated results, revealing the high phase purity and good crystallinity of the obtained MOF. Additionally, the Ni‐DPNDI MOF can be fitted into the “cab” topology type based on theoretical structural simulation and Pawley refinement, with corresponding lattice parameters (*a* = 13.2043 Å, *b* = 21.5517 Å, and *c* = 13.4648 Å; *α* = *γ* = 90°, and *β* = 89.98°), space groups P2/m (Table S1, Supporting Information). Meanwhile, the Pawley refinement PXRD pattern against the experimental result (*R*_p_ = 1.39%, *R*_wp_ = 2.28%) proves the validity of the computational model. In this paddle‐wheel structure, the Ni_2_ active centers are coordinated with four −COOH to construct a stable secondary building unit (SBU) of {Ni_2_(COO)_4_}. After that, the adjoin SBUs are connected by camph^2−^ ligands to form 2D layers with a square extension topology structure. Ultimately, the bipyridine DPNDI acts as pillars that connect these {Ni_2_‐camph_2_} layers to set up a 3D non‐interpenetrating architecture. Moreover, the DPNDI connectors can accelerate charge transfer based on their unique acceptor–donor–acceptor structure and enhance the overlapped level of metal‐ligand orbital, improving the conductivity of Ni‐DPNDI MOF.^[^ ^29^ ^]^ As a result, the Ni‐DPNDI MOF delivers a conductivity of up to 4.63 S m^−1^ at room temperature (Table S2, Supporting Information), which is superior to most reported MOFs (generally in 10^−10^ S m^−1^).^[^ ^30^ ^]^

Additionally, the coordination environment of Ni‐DPNDI MOF was further verified by the FT‐IR and X‐ray photoelectron spectroscopy (XPS) measurements. Compared with the FT‐IR results of the pristine H_2_camph ligand, the peak (1690 cm^−1^) vanished, and two new peaks at 1608 and1402 cm^−1^ appeared in the FT‐IR spectrum of the as‐prepared MOF, which is responding to the asymmetric stretch v_as_(COO) and stretch v_sys_(COO), proving the formation of bidentate bridged carboxylates (Figure S3, Supporting Information). The XPS full‐survey‐scan spectrum illustrates the existence of C, N, O, and Ni elements in the Ni‐DPNDI MOF (Figure S4a, Supporting Information). In the Ni 2p region, the spin‐orbit doublets are situated at 856.1 eV (Ni 2p_3/2_) and 873.7 eV (Ni 2p_1/2_) (with a peak spacing width of 17.6 eV), corresponding to Ni^2+^ in Ni‐DPNDI MOF, and the consequential satellite peaks appear in 861.2 and 879.6 eV (Figure S4b, Supporting Information). The XPS spectrum of N 1s is indexed separately by two peaks, corresponding to pyridinic N‐Ni at 399.9 eV and N─C at 400 eV of Ni‐DPNDI (Figure S4c, Supporting Information). The XPS scan spectrum of O 1s reveals two separate peaks in the range of 528–536 eV, corresponding to the C─O─Ni (532.1 eV) bond and the oxygen in C═O (531.2 eV) of the prepared sample (Figure S4d, Supporting Information).^[^ ^31^, ^32^ ^]^ Moreover, plentiful methyl groups of SBUs that are densely distributed near the Ni nodes, can enhance the chemical stability of Ni‐DPNDI MOF by inhibiting the hydrolytic and rift of the O─Ni bond in an alkali solution. To estimate the stability of Ni‐DPNDI MOF, the powdery samples were soaked in 0.1–5 m KOH electrolyte for three days. The nearly consistent FT‐IR spectra and PXRD patterns of Ni‐DPNDI MOFs suggest good durability of Ni‐DPNDI in KOH electrolyte (Figure S5, Supporting Information). The scanning electron microscope (SEM) pictures illustrate that the Ni‐DPNDI MOFs exhibit a sheet‐like morphology, which is a kind of 2D materials at the micro‐nano dimension (Figure S6, Supporting Information). The porous structure of Ni‐DPNDI MOF was verified by the N_2_ adsorption test at 77 K. The MOF performs the typical type‐IV isotherm, revealing the hierarchical porous property of Ni‐DPNDI. The BET (Brunauer‐Emmett‐Teller) surface area of Ni‐DPNDI MOF is 307 m^2^·g^−1^. Moreover, the micropores and mesopores of Ni‐DPNDI MOF are distributed in 0.4–0.6 and 3–12 nm, respectively. The pore is conducive to the diffusion motion for electrolyte ions (Figure 1d,e). Therefore, the stable and porous Ni‐DPNDI MOF with enhanced electroconductivity was obtained successfully.

To demonstrate its application in the photoelectric field, we further fabricated a large‐area film (100 cm^2^) by facile and low‐cost electrostatic spray deposition (ESD) technology based on homogeneous ink, which was obtained by dispersing Ni‐DPNDI MOF powders in the mixed solution of ethanol and H_2_O with a volume ratio of 1:1 (Figure 1b). In the ESD process, the MOF inks can atomize by overcoming their surface tension with the assistance of an electrical field of 15 kV. Then, the atomized charged mist can deposit the FTO surface to form a film under the assistance of the electrostatic interaction (Figure 1b).^[^ ^33^ ^]^ For assessing the uniformity of the as‐prepared film, we conducted UV–vis spectra of large‐area film at five different sites (A‐E). The transmittance is nearly overlapped in UV–vis spectra at different locations, manifesting the spatial uniformity of the as‐prepared film (Figure 1f). The SEM reveals that Ni‐DPNDI film is constructed by curled nanosheets and its thickness is 251 nm. The SEM mapping of Ni‐DPNDI MOF discloses the existence of C, N, Ni, and O elements, and they are uniformly dispersive around the film (Figure 1h; Figure S7, Supporting Information).

Profiting from the high porosity and unique electronic structure of Ni‐DPNDI MOF, we expect that the MOF film can exhibit excellent electrochemical and EC properties. For estimating the above performance of the Ni‐MOF electrode, the cyclic voltammetry (CV) curve of the Ni‐DPNDI electrode was recorded at a scan rate of 10 mV·s^−1^ in the −1.3 and 0.8 V versus Ag wire and monitored the corresponding spectrum change at 430 nm during the CV process. The CV curve illustrates that Ni‐DPNDI film exhibits an oxidation peak at 0.8 V versus Ag wire and two reduction peaks at 0.28 and −1.0 V versus Ag wire, respectively. This redox characteristic is analogous to our previous work, which further indicated the Ni‐DPNDI MOF owns a similar structure to our reported framework of Ni‐BPY topology with the redox‐active metal center of Ni^2+^.^[^ ^31^ ^]^

**Figure 2:**
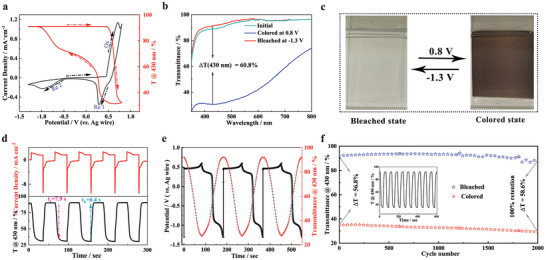
a) CV curve and corresponding transmittance change at 430 nm of Ni‐DPNDI film at a scan rate of 10 smV s^−1^. b) UV–vis spectra of the Ni‐DPNDI film at its initial, colored, and bleached states. c) Optical photographs of Ni‐DPNDI film at colored and bleached states. d) The current densities and in situ transmittance change at 430 nm by switching the cycle of −1.3 and 0.8 V (vs Ag wire) per 30 s. e) The transmittance changes of Ni‐DPNDI electrode at 430 nm during the GCD process at a current density of 1 A g^−1^. f) The cycling stability of Ni‐DPNDI film at colored and bleached states. The above tests are conducted in 0.1 m KOH electrolyte.

In addition, during the anodization process, the color of the Ni‐DPNDI electrode switches from transparent to black‐brown. In the reverse reduction process, the electrode color gradually fades to transparent. Notably, the reversible transmittance change at 430 nm is observed in the CV process, implying the reversible electrochromic property of Ni‐DPNDI film (**Figure** **2**a). To further quantitatively assess the changes of transmittance at the visible region of the Ni‐DPNDI film, we monitored the transmittance change (300–800 nm) of the electrode at its initial, coloration, and bleaching states. The large optical transmittance of over 90% in the initial state reveals the transparency of pristine Ni‐DPNDI MOF film. Upon applying a potential of 0.8 V (vs Ag wire), the transmittance of the Ni‐DPNDI electrode is sharply decreased, accompanying the color change of the film from transparent (initial state) to black‐brown (colored state). Upon the excitation of −1.3 V (vs Ag wire), the transmittance returned to that of the initial state, and the black‐brown film gradually recovered to its initial transparent state. The optical modulation (Δ*T*) is denoted as the difference of EC electrode transmittance at its bleached state (*T*_b_) and colored state (*T*_c_) (Δ*T* = *T*_b_ – *T*_c_), which is an important parameter for EC performance assessment. For smart windows or display applications, the bigger the better. For Ni‐DPNDI film, the maximum of Δ*T* is 60.8% at 430 nm, which surpasses most reported EC‐MOFs.^[^ ^6^, ^31^, ^34^ ^]^ Importantly, the Ni‐DPNDI film realizes neutral coloration (black), which is more suitable for smart windows and the new generation of electronic displays (Figure 2b,c, Table S2, Supporting Information). Switching speed is another key factor for estimating the EC performance of materials. For the applications in the display and smart window, the faster switch is the better. To estimate the switching speed of the Ni‐DPNDI MOF film, the dynamic transmittance change curve (430 nm) was recorded in a chronoamperometry (CA) process under the alternating potentials of 0.8 V and −1.3 V (vs Ag wire) per 30 s. The coloration time (*t*_c_) and the bleaching time (*t*_b_) are determined by the consumed time for EC films to complete 90% of the Δ*T*. The *t*_c_ and *t*_b_ are 7.9 and 6.4 s, respectively. This response speed is comparable with those of most reported EC MOFs and NiO films (Figure 2d; Tables S3 and S4, Supporting Information).^[^ ^35^, ^36^, ^37^ ^]^ In addition, the Ni‐DPDNI film reveals similar EC performance (Δ*T* = 61.7%, *t*_c_ = 7.4 s; *t*_b_ = 10.1 s) in 1 M KOH electrolyte, illustrating that the EC Ni‐DPNDI film can work in more concentration electrolyte (Figures S8 and S9, Supporting Information). Coloration efficiency (CE) can be described as the variations of optical density (ΔOD) induced by the charge density (ΔQ) of EC film during the coloration process. The corresponding relationship is illustrated as the following formula:^[^ ^5^ ^]^

(1) $$\mathrm{CE}\left(\lambda \right)=\Delta \mathrm{OD}/\Delta Q=\log\left(T_{b}/T_{c}\right)/\Delta Q$$

In which λ is the selected wavelength for max Δ*T*. According to the ΔOD/(ΔQ) curve, the CE of Ni‐DPNDI film is 28.9 cm^2^·C^−1^ at 430 nm (Figure S10, Supporting Information). Moreover, the Ni‐DPNID film exhibits an excellent EC performance with an optical modulation of up to 61.8% and a fast response time of within 10 s when using air as background (Figure S11, Supporting Information). Additionally, after powering off for 3600 s, the color of the Ni‐DPNDI film is clearly visible and the transmittance decayed 20%, indicating that Ni‐DPNDI film can maintain its coloring state for over 1 h without any energy consumption (Figure S12, Supporting Information). Moreover, the above phenomenon can be repeated by applying a lower energy of 27.1 mC cm^−2^.

In view of the electrochromism and energy storage triggered by a similar redox reaction, we also accessed the energy storage property of the Ni‐DPNDI MOF film via galvanostatic charge/discharge (GCD) measurement at 1 A·g^−1^ and recorded dynamic spectrum change at 430 nm (Figure 2e). As expected, the capacity of colored Ni‐DPNDI film is 25.3 mAh·g^−1^. Additionally, in the charging process, the film color changes from transparent to black‐brown accompanied by a gradual decrease in transmittance. During the reverse discharging process, the colored film fades away until the transparent state. The above results demonstrate that energy storage levels can be effectively identified according to the color change of the film. The above excellent electrochemical performance makes Ni‐DPNDI MOFs integrate electrochromism and energy storage. More importantly, the Δ*T* value of the Ni‐DPNDI electrode is still well‐remained even after subjecting to 2000 cycles of coloration and bleaching process, surpassing most reported EC MOF materials of our knowledge and even some Ni‐based oxides (Tables S2 and S4, Supporting Information).^[^ ^5^, ^38^, ^39^ ^]^ Moreover, after cycling the 1000 times of coloration and bleaching process, there are not surface cracks on the Ni‐DPNDI film, further proving the good stability of Ni‐DPNDI film (Figure S13, Supporting Information). In comprehensive contrast with other EC MOFs, Ni‐DPNDI MOF delivers an excellent electrochromic property, which has a unique and fast color switch between colorless and black‐brown (Δ*T* = 60.8%, *t*_c_ = 7.9 s; *t*_b_ = 6.4 s) and long‐term life over 2000 cycles (Table S2, Supporting Information). Besides the EC function, the Ni‐DPNDI MOF film possesses the energy storage function concurrently.

**Figure 3:**
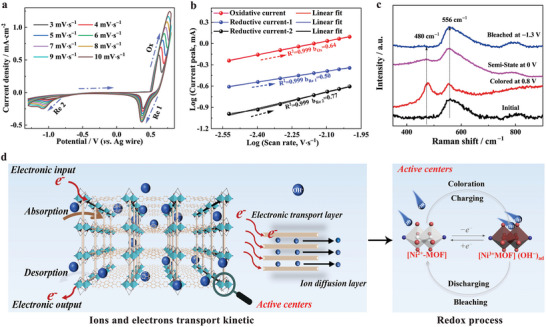
Electrochromic energy storage mechanism of Ni‐DPNDI electrode. a) The CV curves of Ni‐DPNDI electrode at the different scan rates of 3–10 mV s^−1^ in 0.1 m KOH electrolyte. b) The relationship of anodic and cathodic current peak versus scan rate for the Ni‐DPNDI film. The inset is the b value. c) Raman spectra of Ni‐DPNDI film in a 0.1 m KOH electrolyte at initial state, colored state, and stepwise bleached states. d) Schematic diagram of the electrochromic energy storage mechanism for Ni‐DPNDI MOF.

For analyzing the electrochemical kinetics of the Ni‐DPNDI electrode, the scan rate‐dependent CV tests were performed in the range of 3–10 mV s^−1^ during the above electrochemical process (**Figure** **3**a). The current intensity is enhanced as the scan rate increases, which delivers a slight polarization, illustrating the fast electrochemical kinetics of the Ni‐DPNDI film. Furthermore, we qualitatively verified the electrochemical behavior of the Ni‐DPNDI film during the electrochemical reaction kinetics process, which can be described as the formula:^[^ ^40^ ^]^

(2) $$i=a\nu^{b}$$

(3) $$\log\left(i\right)=\log\left(a\right)+\mathrm{blog}\left(\nu \right)$$

where i denotes current intensity at anodic and cathodic peaks. The ν is the scan rate. The a and b are tunable parameters. In addition, the charge‐controlled behavior during the electrochemical process can be judged according to the b parameter. The b = 1 illustrates the surface capacitance‐controlled electrochemical behavior. While the b‐value is 0.5, the electrochemical process is controlled by the diffusion behavior within the active materials. The b‐value falls in between, implying the above two electrochemical behavior co‐existence during the electrochemical process. For the Ni‐DPNDI film, the resulting b‐value during the electrochemical process is ≈0.64 (b_Ox_), 0.50 (b_Re 1_), and 0.77 (b_Re 2_), respectively. It reveals that the oxidation process and the first step of the reduction process for the Ni‐DPNDI electrode are dominated or controlled by diffusion behavior, and the second‐step reduction process exhibits surface‐capacitance‐dominated electrochemical behavior (Figure 3b). The Raman spectroscopy was further used to clarify the reactive sites of the Ni‐DPNDI MOF electrode during electrochemical redox processes (Figure 3c). Compared with that of the initial state, one new peak appears at 480 cm^−1^ in the Raman spectrum after being charged to the black‐brown state (colored state), which is attributed to the Ni‐O vibrations of Ni^3+^ species. Subsequently, the peak gradually vanished at the stepwise bleached processes, and the spectrum recovered to its initial state.^[^ ^31^ ^]^ The Raman results illustrate that the transformation of Ni^3+^ and Ni^2+^ is accompanied during the EC process of Ni‐DPNDI MOFs. In addition, the in situ electrochemical UV–vis spectra illustrate that the DPDNI ligand exists at its neutral state during the EC process (Figure S14, Supporting Information).^[^ ^5^, ^20^, ^22^ ^]^ Moreover, no obvious redox peaks appeared in the CV curve of Ni‐DPNDI film in the range of −1.3–0 V versus Ag/Ag^+^ and its transmittance did not change (Figure S15, Supporting Information), further proving that the EC performance was not induced by the ligands. Additionally, the PXRD patterns of Ni‐DPNDI film at different coloration states prove that the reversible crystal phase transform is accompanied by its EC process (Figure S16, Supporting Information).^[^ ^41^, ^42^, ^43^ ^]^According to the above analysis, the electrochromic energy storage mechanism of Ni‐DPNDI MOF electrode is induced by reversible redox of metal nodes due to fast kinetic processes of the diffusion and capacitance‐coexistence electrochemical behavior.

**Figure 4:**
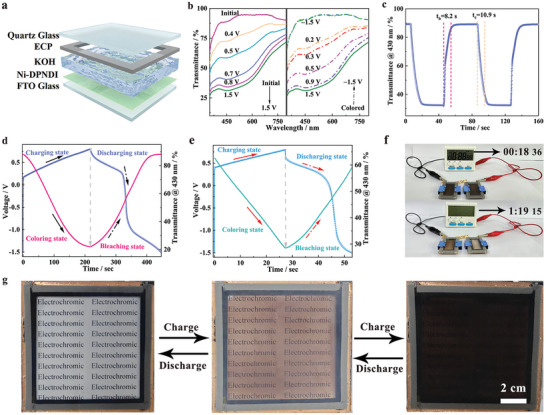
a) Schematic diagram of electrochromic energy storage device (EESD) with Ni‐DPNDI electrode as the working layer, ECP as the counter layer, and 0.1 m KOH solution as an electrolyte. b) The UV–vis spectra under different voltages. c) The in situ transmittance response at 430 nm by cycling of 1.8 V and −1.5 V per 40 s. GCD curve and the corresponding transmittance change at 430 nm at different current densities of d) 1 A·g^−1^ and e) 4 A·g^−1^. f) The display of EESD connected in series for powering the digital watch. g) Color change photographs of a 100 cm^2^ EESD during the charge and discharge processes.

According to the excellent electrochemical and optical properties of Ni‐DPNDI, electrochromic energy storage devices (EESD) were assembled, using Ni‐DPNDI film, etched carbon paper (ECP), and 0.1 m KOH electrolyte solution. ECP was employed as the ion‐storage layer due to its high conductivity and superior ability to balance electric charges (Figures S17 and S18, Supporting Information).^[^ ^44^ ^]^ The assembled device reveals a reversible color change between transparent and neutral coloration (black‐brown) in the electrochemical process. The device achieves a large modulation of 59% at 430 nm, a rapid switching speed (*t*_c_ = 10.9 s; *t*_b_ = 8.2 s), and an enhanced coloration efficiency (37 cm^2^·C^−1^) (**Figure** **4**a–c; Figures S19–21, Supporting Information). In addition, compared to Ni‐DPNDI // FTO device ((Δ*T* = 50.2%, *t*_c_ = 30.9 s; *t*_b_ = 60.3 s), the Ni‐DPNDI // ECP device delivers significant advantage at optical modulation and switch speed, illustrating a good effect of ECP counter electrode in the EC device (Figure S22, Supporting Information). Additionally, the Ni‐DPNDI // ECP device can be recovered to its transparent state at a lower applied voltage. Moreover, the device exhibits a stability of 400 cycles with no attenuation, which is enhanced than most reported EC MOF devices (Figure S23 and Table S3, Supporting Information).^[^ ^21^ ^]^ Moreover, the EESD can work in a 1 m KOH electrolyte and the EESD delivers a modulation of 58.2%, a fast response time of within 10 s, and a coloration efficiency of 34.7 cm^−2^ C^−1^, proving that the EESD can work in more demanding scenarios (Figure S24, Supporting Information). Additionally, the specific capacity of the EESD was 27.8, 21.5, 18.3, 15.9, and 14 mAh·g^−1^ at different current densities of 1–4 A·g^−1^. Moreover, there is a clear transmittance change during the GCD process. The EESD remained over 50% of its initial optical modulation at a higher current density of 4 A·g^−1^, further proving the superior rate capability of the device (Figures 4d,e; Figure S25, Supporting Information). Two EESD at coloration state in series can drive the watch working for over 60 minutes, illustrating a good effect of energy storage of the device (Figure 4f; Video S1, Supporting Information). Besides, we constructed a large‐area electrochromic energy storage smart window (100 cm^2^) to highlight the practical application of Ni‐DPNDI. The obtained smart window delivers dynamical color regulation, and efficient energy storage performance (Figure 4g; Figure S26, Supporting Information).

## 3 Conclusion

To conclude, we presented a Ni‐DPNDI metal‐organic framwork (MOF) with enhanced electronic conductivity and robust chemical stability in electrolytes. The excellent electrochemical performance makes the MOF integrate the electrochromic and energy storage performance into one electrode. As the bifunctional electrode material, the Ni‐DPNDI MOF exhibits a spacious optical modulation of 60.8%, a rapid switching time within 10 s, an outstanding cycling lifetime over 2000 cycles, and a commensurate specific capacitance (25.3 mAh·g^−1^). Moreover, the coloration state of the Ni‐DPNDI electrode can reflect energy levels in real‐time. Furthermore, a large area (100 cm^2^) electrochromic energy storage smart window based on Ni‐DPNDI MOF was constructed to demonstrate its promising application. Consequently, the dual‐functional MOF with electrochromic and energy storage characteristics presented here could become promising candidates for new‐generation transparent display materials for electronics of diverse applications.

## Conflict of Interest

The authors declare no conflict of interest.

## Supporting information



Supporting Information

Supplemental Video1

## Data Availability

The data that support the findings of this study are available in the supplementary material of this article.

## References

[1] K. T. Keasler , M. E. Zick , E. E. Stacy , J. Kim , J. H. Lee , L. Aeindartehran , T. Runcevski , P. J. Milner , Science 2024, 381, 1455. 10.1126/science.adg8835 PMC10799685 37769097

[2] D. L. Perl , S. J. Lee , A. Ferguson , G. B. Jameson , S. G. Telfer , Nat. Chem. 2023, 15, 1358. 37537296 10.1038/s41557-023-01277-z

[3] J. Liang , Y. Ma , Y. Li , W. Zhang , H. Hu , J. Su , Z. Yao , W. Gao , W. Shang , T. Deng , J. Wu , Nano Lett. 2024, 24, 7293. 10.1021/acs.nanolett.4c01278 38843402

[4] D. Mücke , I. Cooley , B. K. Liang , Z. Wang , S. Park , R. Dong , X. Feng , H. Qi , E. Besley , U. Kaiser , Nano Lett. 2024, 24, 3014. 38427697 10.1021/acs.nanolett.3c04125 PMC10941249

[5] A. Kumar , J. Li , A. K. Inge , S. Ott , ACS Nano 2023, 17, 21595. 37851935 10.1021/acsnano.3c06621 PMC10655172

[6] X. Fan , S. Wang , M. Pan , H. Pang , H. Xu , ACS Energy Lett. 2024, 9, 2840.

[7] S. Cheng , Z. Cao , Y. Liu , J. Zhang , L. Cavallo , E. Xie , J. Fu , Energ. Environ. Sci. 2024, 17, 1997.

[8] Y. Xu , L. Jiao , J. Ma , P. Zhang , Y. Tang , L. Liu , Y. Liu , H. Ding , J. Sun , M. Wang , Z. Li , H. L. Jiang , W. Chen , Joule 2023, 7, 515.

[9] C. N. Hong , A. B. Crom , J. I. Feldblyum , M. R. Lukatskaya , Chem 2023, 9, 798.

[10] C. N. Hong , A. B. Crom , J. I. Feldblyum , M. R. Lukatskaya , J. Am. Chem. Soc. 2020, 142, 12367.32532157

[11] J. Y. Choi , J. Flood , M. Stodolka , H. T. B. Pham , J. Park , ACS Nano 2022, 16, 3145. 35119816 10.1021/acsnano.1c10838

[12] M. Ding , X. Cai , H. Jiang , Chem. Sci. 2019, 10, 10209. 32206247 10.1039/c9sc03916c PMC7069376

[13] H. Min , O. Kwon , J. E. Lee , J. Kim , N. Lee , K. Eum , K. Lee , D. Kim , W. Lee , Adv. Mater. 2024, 36, 2309041. 10.1002/adma.202309041 38041566

[14] H. Liang , R. Li , C. C. Li , Y. Li , Q. Zhang , H. Wang , Mater. Horiz. 2019, 6, 571.

[15] K. Chen , Z. Sun , R. Fang , Y. Shi , H. Cheng , F. Li , Adv. Funct. Mater. 2018, 28, 1707592.

[16] J. Liu , X. Song , T. Zhang , S. Liu , H. Wen , L. Chen , Angew. Chem., Int. Ed. 2021, 60, 5612. 10.1002/anie.202006102 32452126

[17] M. L. Aubrey , B. M. Wiers , S. C. Andrews , T. Sakurai , S. E. Reyes‐Lillo , S. M. Hamed , C. Yu , L. E. Darago , J. A. Mason , J. O. Baeg , F. Grandjean , G. J. Long , S. Seki , J. B. Neaton , P. D. Yang , J. R. Long , Nat. Mater. 2018, 17, 625. 29867169 10.1038/s41563-018-0098-1

[18] S. S. Shinde , C. H. Lee , J. Jung , N. K. Wagh , S. H. Kim , D. H. Kim , C. Lin , S. U. Lee , J. H. Lee , Energy Environ. Sci. 2019, 12, 727.

[19] Z. Xia , X. Jia , X. Ge , C. Ren , Q. Yang , J. Hu , Z. Chen , J. Han , G. Xie , S. Chen , Angew. Chem., Int. Ed. 2021, 60, 10228. 10.1002/anie.202100123 33474801

[20] C. R. Wade , M. Li , M. Dincă , Angew. Chem., Int. Ed. 2013, 52, 13377. 10.1002/anie.201306162 24133021

[21] K. AlKaabi , C. R. Wade , M. Dincă , Chem 2016, 1, 264.

[22] R. Li , K. Li , G. Wang , L. Li , Q. Zhang , J. Yan , Y. Chen , Q. Zhang , C. Hou , Y. Li , H. Wang , ACS Nano 2018, 12, 3759. 29595953 10.1021/acsnano.8b00974

[23] Z. Lu , R. Li , L. Ping , Z. Bai , K. Li , Q. Zhang , C. Hou , Y. Li , W. Jin , X. Ling , H. Z. Wang , Cell Rep. Phys. Sci. 2022, 3, 100866.

[24] C. Kung , T. Wang , J. E. Mondloch , D. Fairen‐Jimenez , D. M. Gardner , W. Bury , J. M. Klingsporn , J. C. Barnes , R. Van Duyne , J. F. Stoddart , M. R. Wasielewski , O. M. Farha , J. T. Hupp , Chem. Mater. 2013, 25, 5012.

[25] D. Feng , T. Lei , M. R. Lukatskaya , J. Park , Z. Huang , M. Lee , L. Shaw , S. Chen , A. A. Yakovenko , A. Kulkarni , J. Xiao , K. Fredrickson , J. B. Tok , X. Zou , Y. Cui , Z. Bao , Nat. Energy 2018, 3, 30.

[26] E. M. Miner , T. Fukushima , D. Sheberla , L. Sun , Y. Surendranath , M. Dincă , Nat. Commun. 2016, 7, 10942. 26952523 10.1038/ncomms10942 PMC4786780

[27] S. Guha , F. Goodson , R. J. Clark , S. Saha , CrystEngComm 2012, 14, 1213.

[28] S. Guha , S. Saha , J. Am. Chem. Soc. 2010, 132, 17674. 21114330 10.1021/ja107382x

[29] R. Ji , Y. Dong , X. Sun , P. Li , R. Zhang , C. Pan , H. Zhao , Y. Zhu , Adv. Energy Mater. 2024, 14, 2401437.

[30] Y. Zhou , L. Hang , Coordin. Chem. Rev. 2021, 430, 213665.

[31] J. Feng , X. Wang , Y. Luo , J. Wang , Z. Wang , C. Y. Wei , G. Cai , ACS Appl. Mater. Interfaces 2024, 16, 1170. 38149966 10.1021/acsami.3c16801

[32] G. Nagaraju , S. C. Sekhar , B. Ramulu , S. K. Hussain , D. Narsimulu , J. Yu , Nano–Micro Lett. 2020, 13, 17. 10.1007/s40820-020-00528-9 PMC8187485 34138181

[33] J. Kim , W. Chung , K. Kim , D. Y. Kim , K. Paeng , S. M. Jo , S. Jang , Adv. Funct. Mater. 2010, 20, 3538.

[34] L. Pan , R. Li , C. Zhang , Z. Lu , K. Li , Q. Zhang , C. Hou , Y. Li , H. Wang , ACS Appl. Electron. Mater. 2022, 4, 2915.

[35] J. Wang , R. Zhu , Y. Gao , Y. Jia , G. Cai , J. Phys. Chem. Lett. 2023, 14, 2284. 36826414 10.1021/acs.jpclett.3c00050

[36] X. Wu , K. Wang , J. Lin , D. Yan , Z. Guo , H. Zhan , J. Colloid. Interf. Sci. 2021, 594, 73. 10.1016/j.jcis.2021.02.083 33756370

[37] D. Mohanadas , T. B. S. A. Ravoof , Y. Sulaiman , Sol. Energ. Mat. Sol. C 2020, 214, 110596.

[38] A. Mazel , L. Rocco , N. Penin , A. Rougier , Adv. Opt. Mater. 2023, 11, 2202939.

[39] J. Liu , X. Y. D. Ma , Z. Wang , L. Xu , T. Xu , C. He , F. Wang , X. Lu , ACS Appl. Mater. Interfaces 2020, 12, 7442. 31958011 10.1021/acsami.9b20388

[40] S. Liu , C. Wei , H. Wang , W. Yang , J. Zhang , Z. Wang , W. Zhao , P. S. Lee , G. Cai , Nano Energy 2023, 110, 108337.

[41] F. Su , F. F. Xing , X. Wang , F. Y. Liu , L. Z. Zhang , Z. S. Wu , Energy Environ. Sci. 2023, 16, 222.

[42] J. Zhu , H. Shen , X. Shi , F. Yang , X. Hu , W. Zhou , H. Yang , M. Gu , Anal. Chem. 2019, 91, 11055. 31368303 10.1021/acs.analchem.9b01571

[43] C. K. Chan , H. Peng , R. D. Twesten , K. Jarausch , X. F. Zhang , Y. Cui , Nano Lett. 2007, 7, 490. 17256918 10.1021/nl062883j

[44] Y. Luo , H. Jin , Y. Lu , Z. Zhu , S. Dai , L. Huang , X. Zhuang , K. Liu , L. Huang , ACS Energy Lett. 2022, 7, 1880.

