# Supplementary material for: Alkali‐Stable Metal–Organic Frameworks with Enhanced Electroconductivity for Black‐Brown Electrochromic Energy Storage Smart Window
Source: Adv Sci (Weinh). 2024 Oct 1;11(44):2407297. doi: 10.1002/advs.202407297 (PMC11600288; doi:10.1002/advs.202407297)
Supplement: Supplementary file 1 — Supporting Information [file ADVS-11-2407297-s002.docx]

Supporting Information

Alkali-stable Metal-Organic Frameworks with Enhanced Electroconductivity for Black-Brown Electrochromic Energy Storage Smart Window

Xinyi Wang^#^, Zhiqiang Liu^#^, Heqi Ma, Yiwen Liu, Qing Sui, Jifei Feng*, and Guofa Cai*

^#^Xinyi Wang and ZhiQiang Liu have equal contributions to this work.

*E-mail: jffeng@henu.edu.cn; caiguofa@henu.edu.cn

**Experimental Procedures**

**1.1. Materials**

4-Aminopyridine (Apy, >99.0%), 1,4,5,8-Naphthalenetetracarboxylic dianhydride (NDI, 98.0%) were purchased from Energy Chemical Reagent company. DL-(±)-camphoric acid (H_2_camph, 99%, Adamas) and Polyvinylpyrrolidone (PVP, Adamas) were brought from the Tansoole company. N, N-dimethylformamide (DMF, 99.9%) was purchased by Aladdin company. Nickel (II) nitrate hexahydrate (Ni(NO_3_)_2_·6H_2_O, ≥98.0%), acetone (99.5%), and ethanol (EtOH, 99.7%) were purchased from Sinopharm Chemical Reagent Co. Ltd (China). Potassium hydroxide (KOH, ≥ 85%) was purchased from Kermel Chemical Reagent. Above all chemicals and reagents were used without further purification. Fluorine-doped tin oxide (SnO_2_: F, FTO) transparent conductive glasses (14 Ω sq^−1^ cm^-2^, thickness: 1.6 mm) were purchased from Yingkou PV∙Tech New Energy Technology Co., Ltd. Deionized water (18.25 MΩ·cm^-1^) was obtained from Ulupure equipment (UPR-11-10T, 220 V, ~ 50 Hz, 100 W).

**1.2. Synthesis of** **2,7-Di(Pyridin-4-Yl)Benzo[Lmn][3,8]Phenanthroline-1,3,6,8(2H,7H)-Tetraone (DPNDI)**

1,4,5,8-Naphthalenetetracarboxylic dianhydride (NDI, 500 mg, 1.864 mmol), 4-Aminopyridine (Apy, 350 mg, 3.752 mmol), and 15 mL anhydrous DMF were added in a 100 mL double-necked round-bottomed flask. Then, the above system was purged with N_2_ atmosphere and stirred at 450 r·min^−1^ at 130 °C for 18 h. A crystalline solid precipitated during cooling and the pink powder was collected by vacuum suction filtration with DMF and anhydrous ether several times and dried overnight at 60 °C in 70% yield, eventually. ^1^H NMR (500 MHz, DMSO-d^6^, 25 °C): δ = 8.81 (dd, J = 1.6 Hz and 3 Hz, 4 H), 8.75 (s, 4 H), and 7.58 (dd, J = 1.6 Hz and 4.5 Hz, 4 H) ppm. FT-IR (cm-1): 3069.97 (aromatic CH), 1712.27 (amide C=O stretching), 1661.71 (N−C=O imide stretching).

**1.3. Hydrothermal synthesis of Ni-DPNDI MOF**

A mixture of H_2_camph (100 mg, 0.5 mmol), DPNDI (105 mg, 0.25 mmol), Ni(NO_3_)_2_·6H_2_O (150 mg, 0.5 mmol), 120 mg PVP, and DMF (5 mL) was sealed in the screwed-cap Teflon-lined stainless-steel autoclave and heated at 120 ℃ for 2 days. After cooling to room temperature (≈ 25 ℃), the pale green Ni-DPNDI was collected and washed with DMF, EtOH, and acetone three times, respectively. The washed Ni-DPNDI powders dried out in a vacuum at 60 ℃ for 8 h.

**1.4. Preparation of the Ni-DPNDI thin film by electrostatic spraying deposition (ESD) method**

**Preparation of the substrates**

FTO conductive glass was cut into 2.5 × 5 cm^2^ and 10 × 10 cm^2^. Then, the cut glasses were washed in an ultrasonic bath with acetone, deionized water, and EtOH solution for 15 min, respectively. Afterward, the washed glasses dried out with N_2_ before use.

**Preparation of Ni-DPNDI inks**

A homogenous ink with a concentration of 0.125 mg·mL^−1^ was prepared by dispersing 10 mg Ni-DPNDI powders d into a mixed solvent of EtOH and deionized water (80 mL, V/V = 1:1) and stirring at 1200 r·min^−1^ for 4 h at room temperature.

**Preparation of Ni-DPNDI film**

The Ni-DPNDI ink was transferred into an injector with a stainless steel needle (inner diameter: 0.4 mm). The FTO glass is used as the substrate. The distance between the needle and the substrate is 15 cm approximately. During the ESD process, a 15 kV DC voltage was conducted between the needle and substrate. Upon the excitation of a high DC field, the Ni-DPNDI inks were atomized into the aerosol. Then, the Ni-DPNDI aerosol was attracted to the surface of the FTO substrate. Additionally, the spray speed is 1 mL·h^−1^. Simultaneously, a near-infrared (NIR) light was used as the heater source to accelerate the evaporation of solvents. After the evaporation, the Ni-DPNDI film was prepared successfully. Moreover, the films with sizes of 2.5 × 5 cm^2^ and 10 × 10 cm^2^ were obtained by changing the spraying area.

**1.5. Preparation of etched carbon paper (ECP)**

Relative hydrophilicity carbon paper (CP) was oxidized at a constant potential of 2.7 V vs Ag/AgCl for 10 min in a three-electrode system with Ag/AgCl as reference electrode, a graphite sheet as a counter electrode, and 1 M H_2_SO_4_ aqueous solution as electrolyte. After oxidation, the ECP exhibits a higher hydrophilicity, which can increase the contact area with the aqueous electrolyte. Moreover, the etched carbon paper (ECP) displays a low sheet resistance of 442.0 mΩ/sq. Then, the ECP was cut into the rectangular ambulatory plane for the device.

**1.6. Assembly of the electrochromic-energy storage bi-functional devices (EESD)**

In the devices, Ni-DPNDI-FTO films (2.5 × 5 cm^2^ and 10 × 10 cm^2^) were used as the electrochromic layer (work electrode), and a rectangular-ambulatory plane ECP on the quartz glass or FTO glass was employed as the ion storage layer (counter electrode). Then, the space of 1.5 mm between the above electrodes was fixed by adding a mounting tape (VHB 4010, 3 M) between two electrodes. Finally, 0.1 M KOH or 1 M KOH electrolyte was injected into the space.

**1.7. Characterization**

Nuclear Magnetic Resonance Spectrometer (NMR) spectra of DPNDI were recorded on the Bruker AVANCE III HD instrument with a frequency of 500 MHz in the DMSO-d^6^ solution. X-ray diffraction (XRD) tests were performed to investigate the crystal structure of Ni-DPNDI by scanning in the 2θ range of 3-50° on Bruker D8-ADVANCE. The conductivity of Ni-DPNDI MOFs was measured on Quantum Design-PPMS-9 with KEITHLEY 2400 (i= 100 μA) and KEITHLEY2182A at room temperature (300 K). The Brunauer-Emmett-Teller (BET, BEL SORP MAX) measurement was conducted to analyze the pore structure of Ni-DPNDI powder. Fourier transform infrared spectroscopy (FT-IR, FIR STA 8000) was used to demonstrate the structure and chemical bonds of molecules. The microstructure of the samples was investigated by field emission scanning electron microscopy (FESEM, Navo Nano SEM 450). The Raman spectroscopy was collected by the Micro-Raman Spectroscopy System (Raman, Renishaw inVia) for the change of characteristic frequency and vibration mode of the material in diﬀerent states with the excitation wavelength of 532 nm. X-ray photoelectron spectroscopy (XPS, AXIS ULTRA) was used to determine the element valence states of the sample. The electrochemical and electrochromic performance of Ni-DPNDI film were estimated via the in situ spectroelectrochemical method on the electrochemical workstation (AUTOLAB PGSTAT302N) and UV-Vis-NIR spectrophotometer (SHIMADZU UV-3600 plus).

Figures and Tables

**
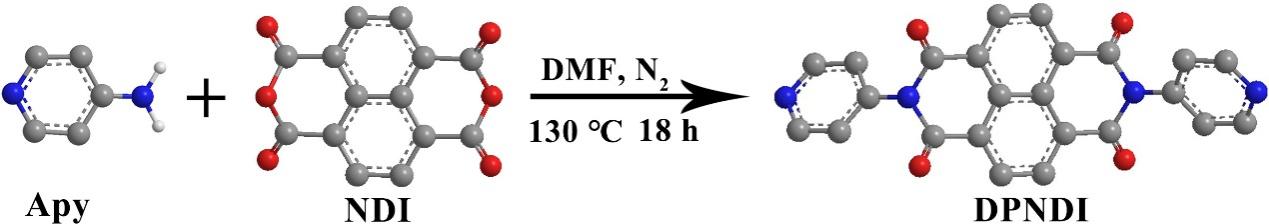
**

**Figure S1.** The synthesis reaction formula of DPNDI ligand.


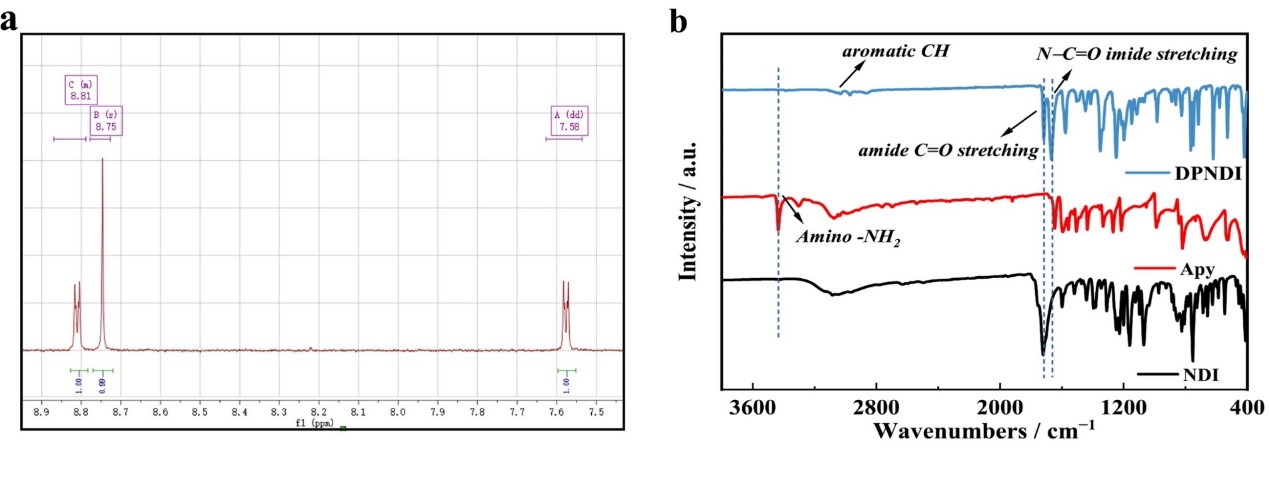


**Figure S2.** (a) The ^1^H NMR spectrum of DPNDI and (b) FT-IR spectra of DPNDI (blue), Apy (red), and NDI (black).


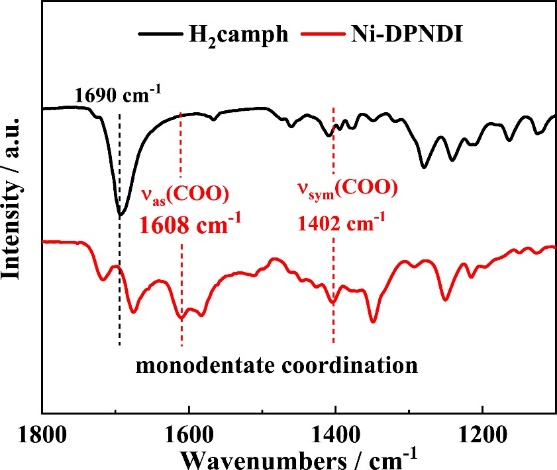


**Figure S3.** The FT-IR spectra of Ni-DPNDI MOF (red) and H_2_camph ligand (black).


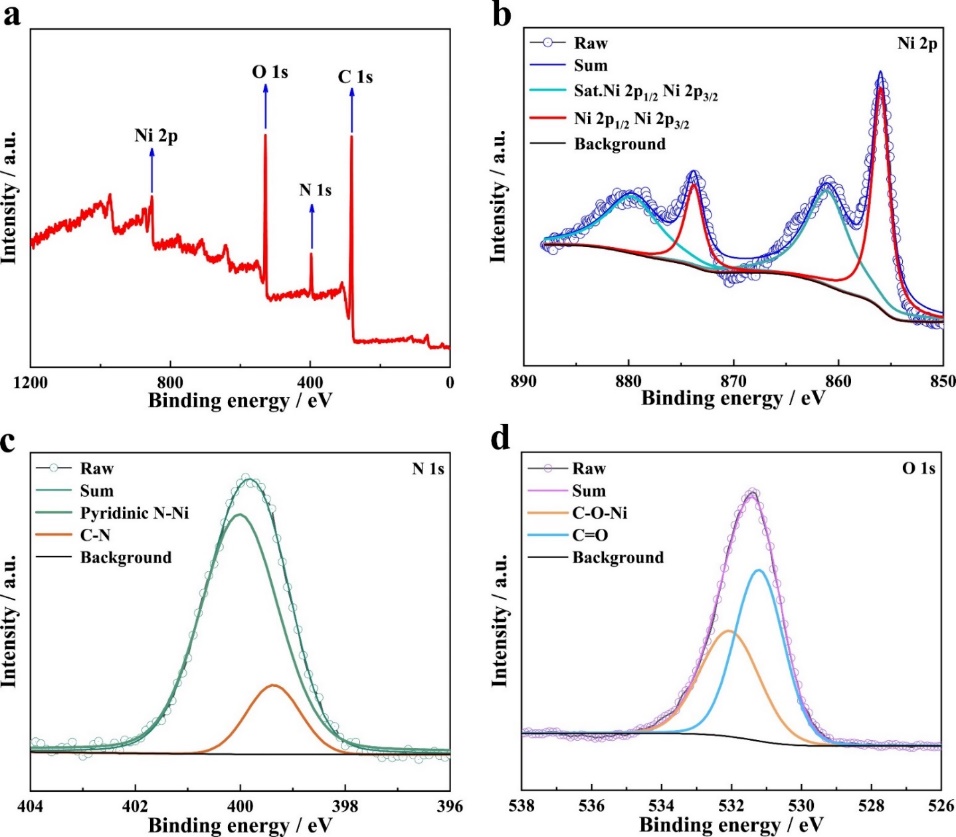


**Figure S4.** (a) The full survey spectrum and (b) the Ni 2p, (c) N 1s, (d) O 1s high-resolution XPS spectra of the Ni-DPNDI powder.


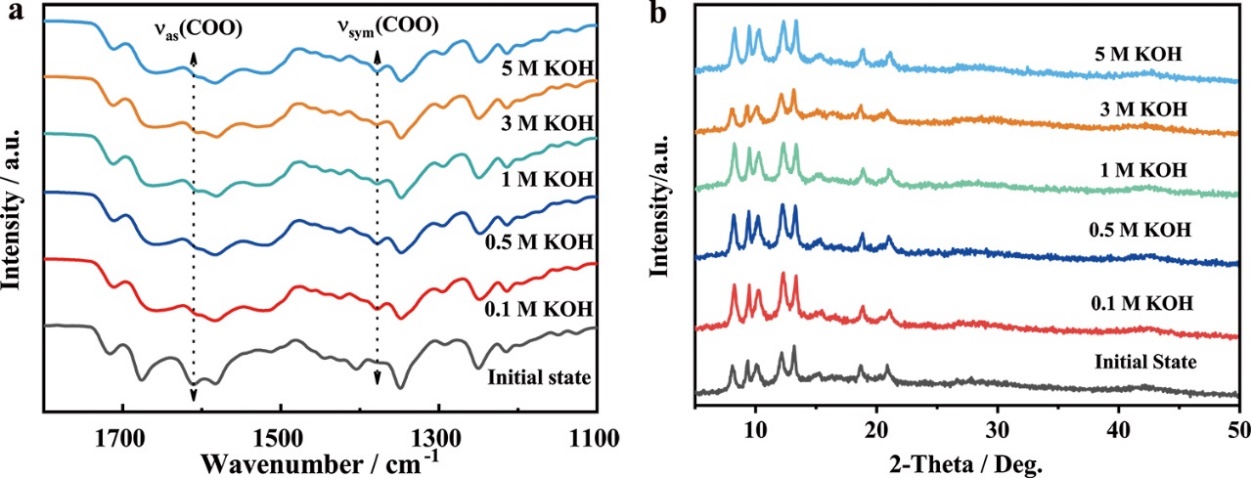


**Figure S5.** The FT-IR spectra (a) and PXRD patterns (b) of Ni-DPNDI powders after immersing in various concentrations of KOH solution for 3 days.


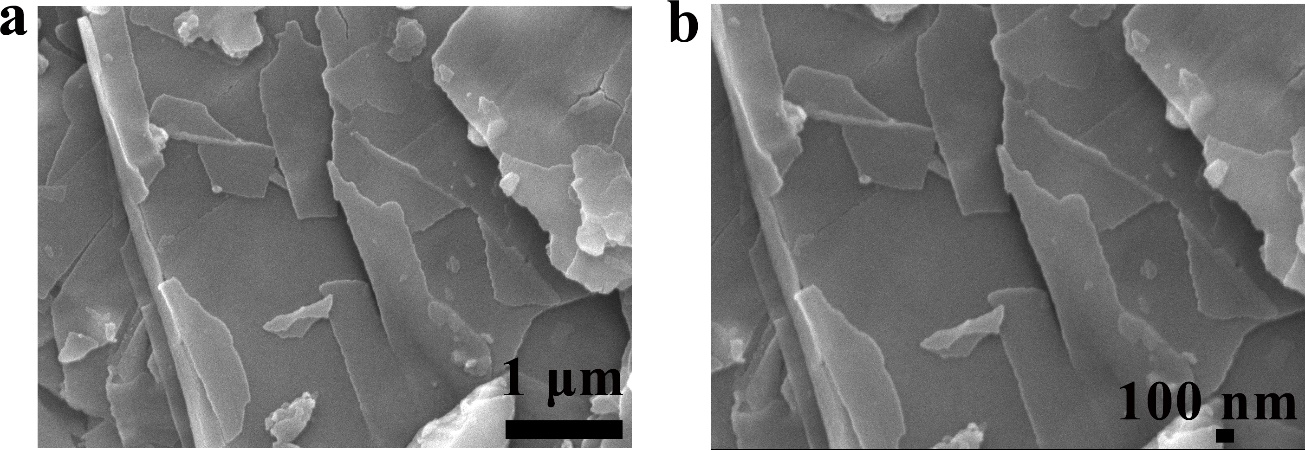


**Figure S6.** SEM image of powdery Ni-DPNDI.

**
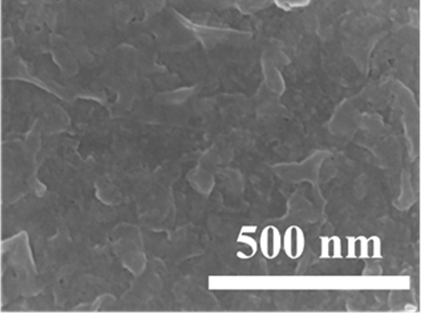
**

**Figure S7.** SEM images of Ni-DPNDI film.

**
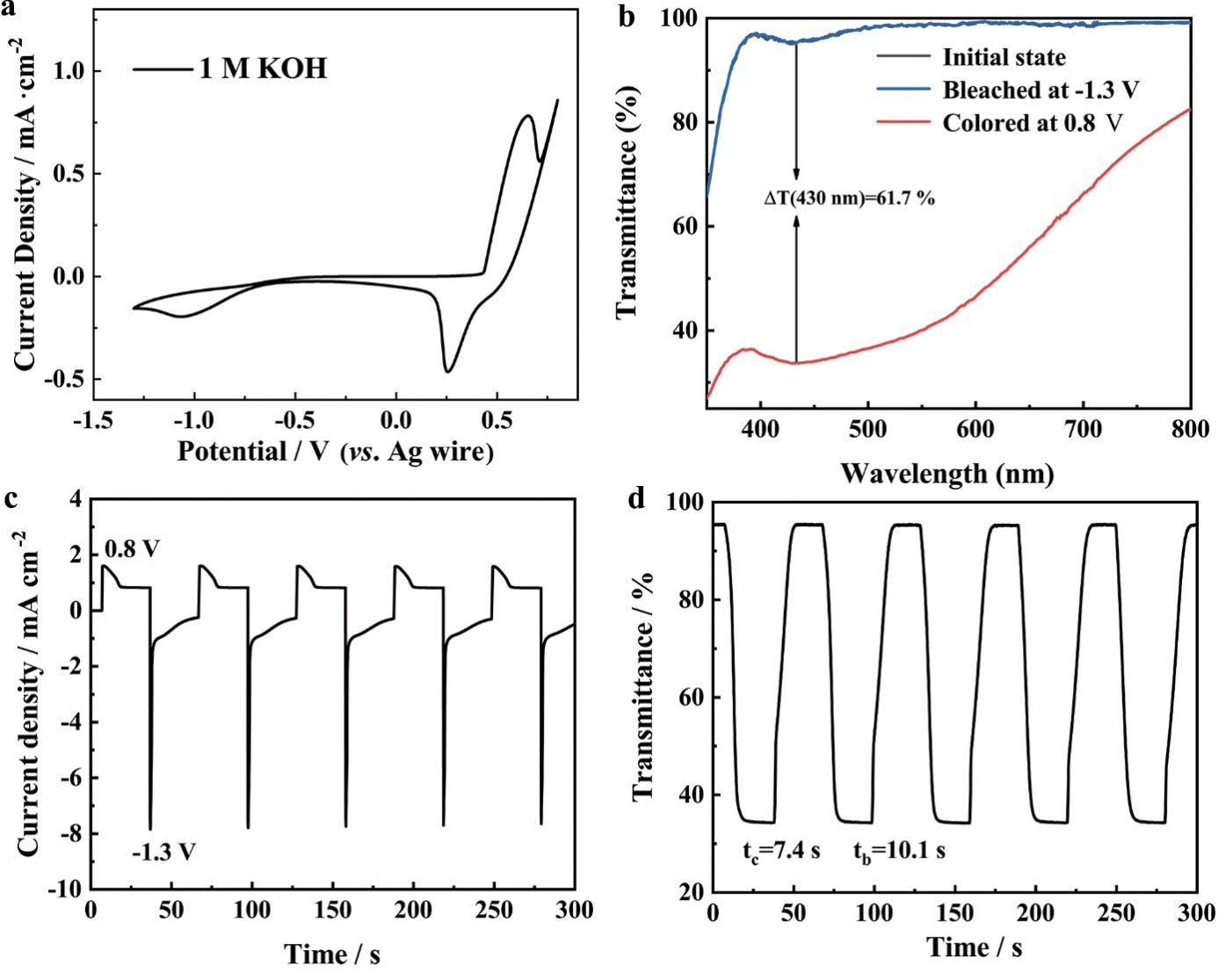
**

**Figure S8** The EC performance of Ni-DPNDI film in 1 M KOH electrolyte. (a). CV curve of Ni-DPNDI film. (b). The UV-vis spectra of Ni-DPNDI film. (c). The diagram of current density change of Ni-DPNDI film by applying the square wave voltage of 0.8 and −1.3 V vs. Ag wire. (d). The in-situ transmittance change of the Ni-DPNDI film at 430 nm.

**
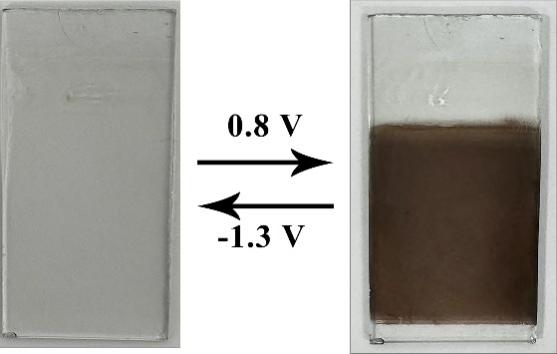
**

**Figure S9.** The EC digital pictures of Ni-DPNDI film at its bleached and colored states in 1 M KOH electrolyte.


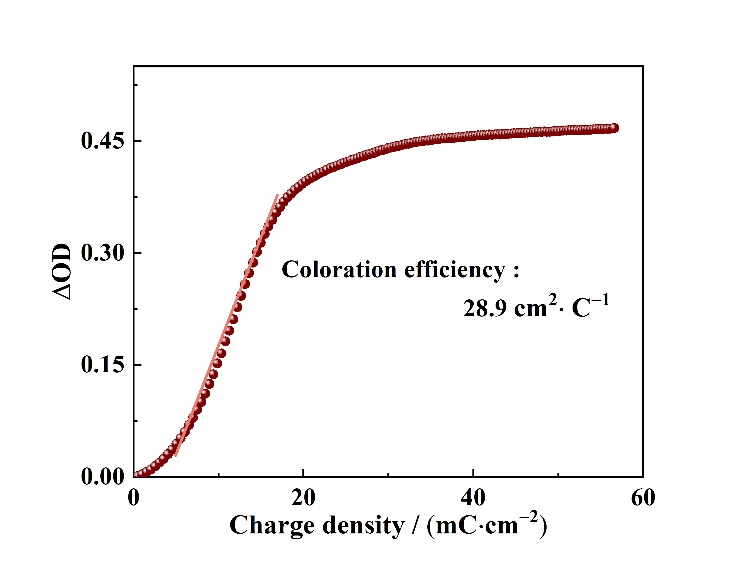


**Figure S10.** The relationship of ΔOD versus charge density. The inset is the coloration efficiency of Ni-DPNDI film.


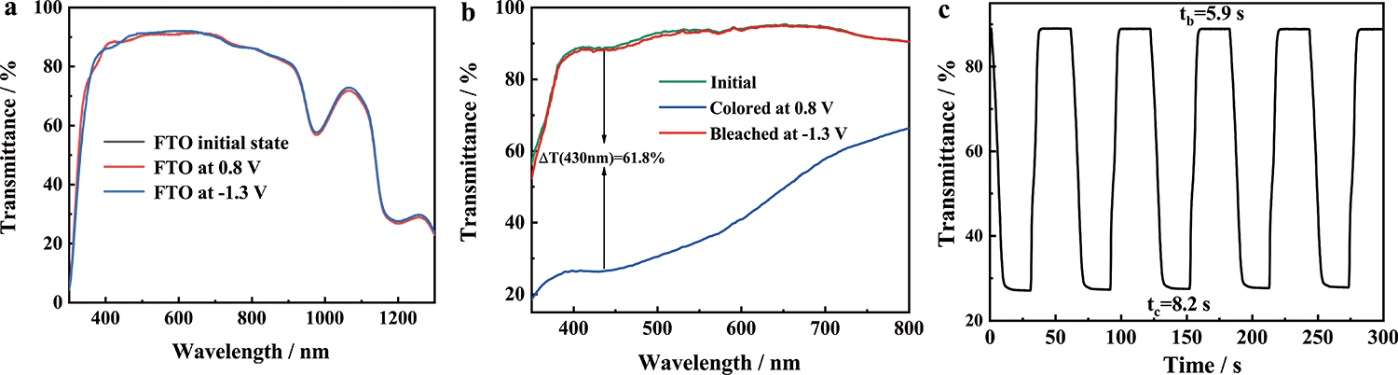


**Figure S11.** The optical performance of Ni-DPNDI film and FTO glass using air as background. (a). The UV-vis spectra of FTO at different applied potentials. (b). The UV-vis spectra of Ni-DPNDI film at its initial, colored, and bleached states. (c). The transmittance change of Ni-DPNDI film at 430 nm by switching the potential cycle of 0.8 and -1.3 V vs Ag wire.


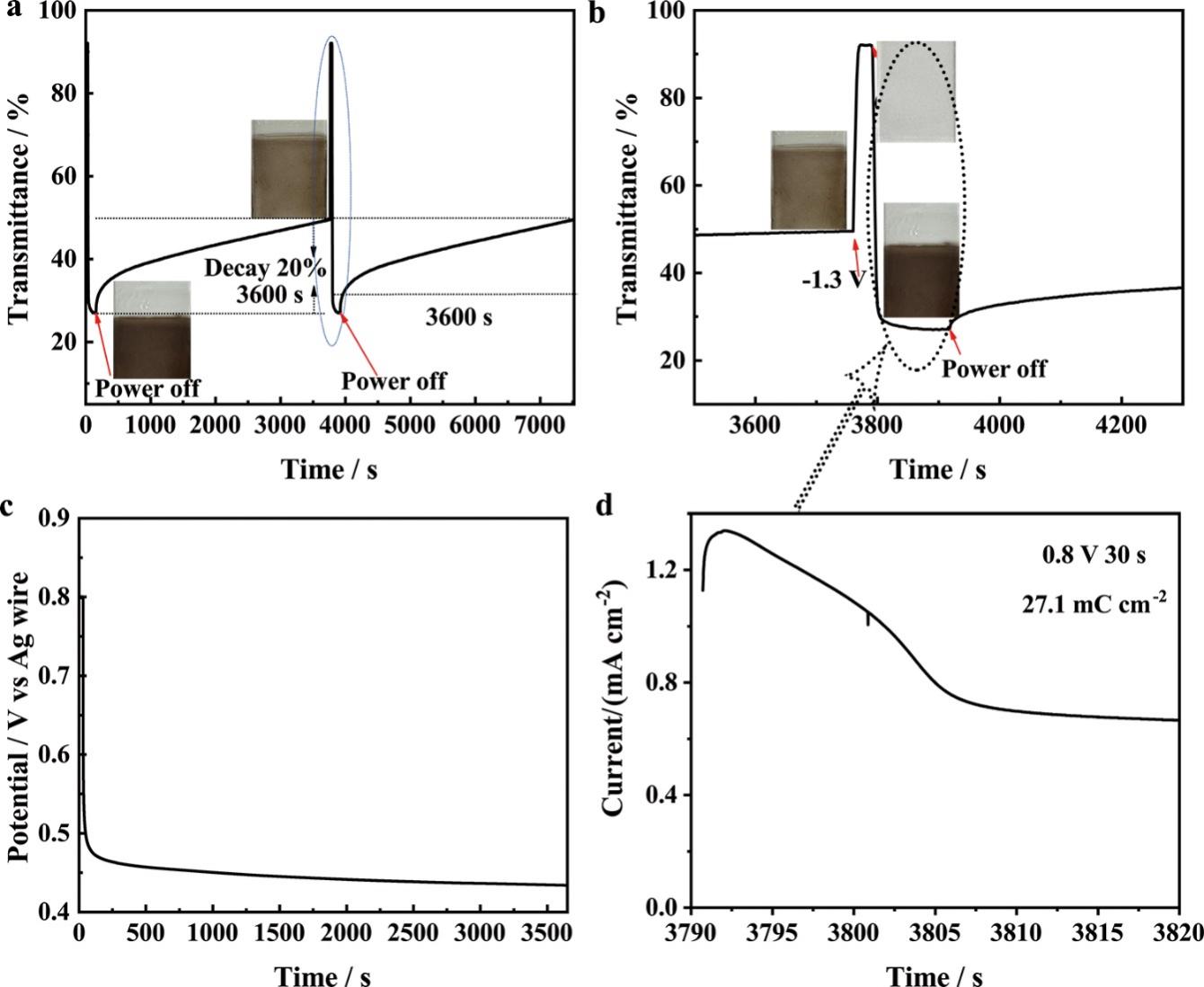


**Figure S12.** (a, b)The transmittance of Ni-DPNDI film at 430 nm after powering off. The inset is the digital pictures of Ni-DPNDI film after powering off at different time. (c) Open-circuit potential change after powering off. (d) The current density changes after applying 0.8 V vs Ag wire.


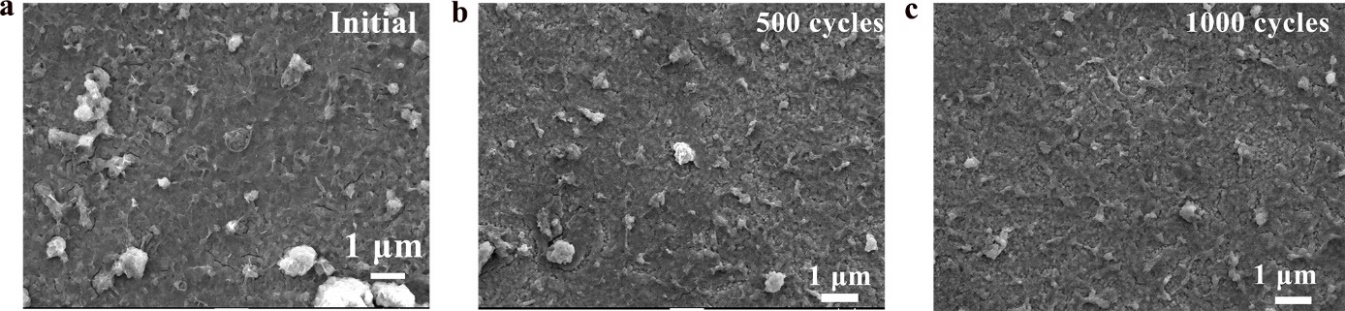


**Figure S13.** SEM images of Ni-DPNDI film before and after the EC test for 500 and 1000 cycles.


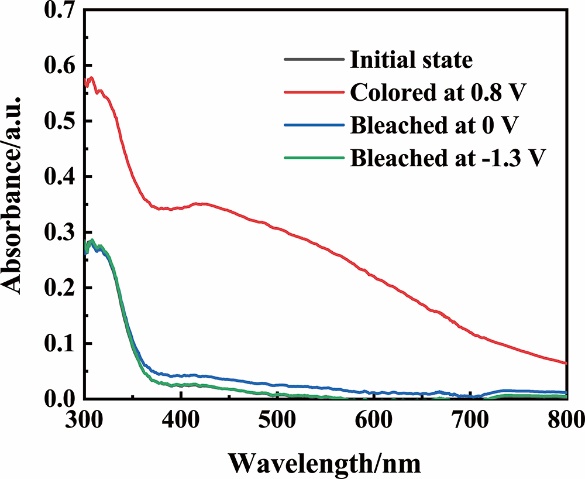


**Figure S14.** UV-vis absorbance spectra of Ni-DPNDI film at different coloration states.


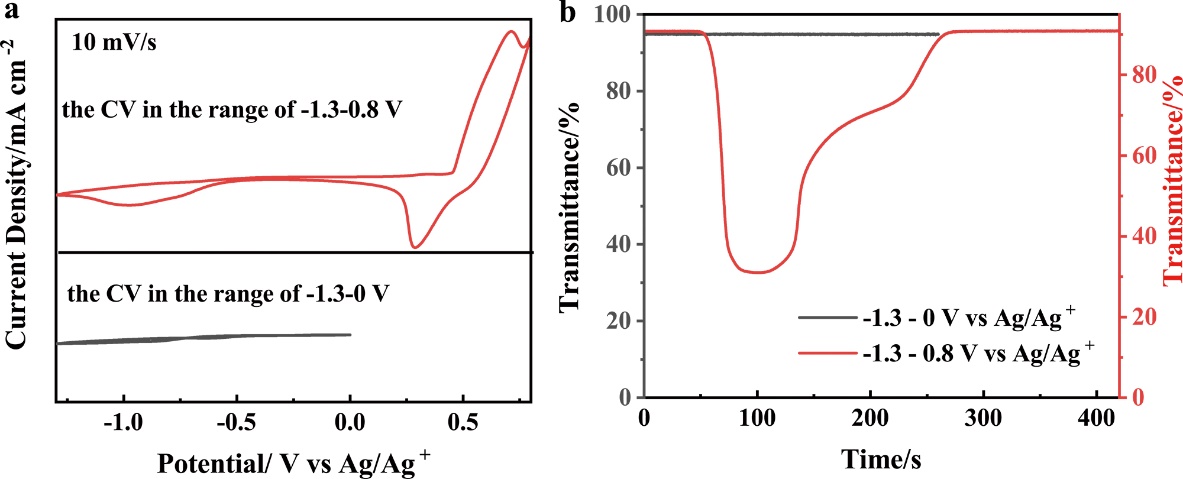


**Figure S15.** (a) CV curves of Ni-DPNDI film at different scanning potential ranges. (b) The corresponding transmittance changes at 430 nm.

**
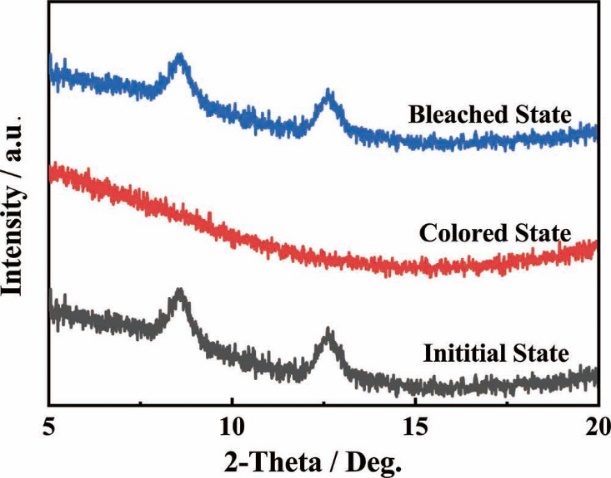
**

**Figure S16.** PXRD patterns of Ni-DPNDI film at its initial, colored and bleached states.


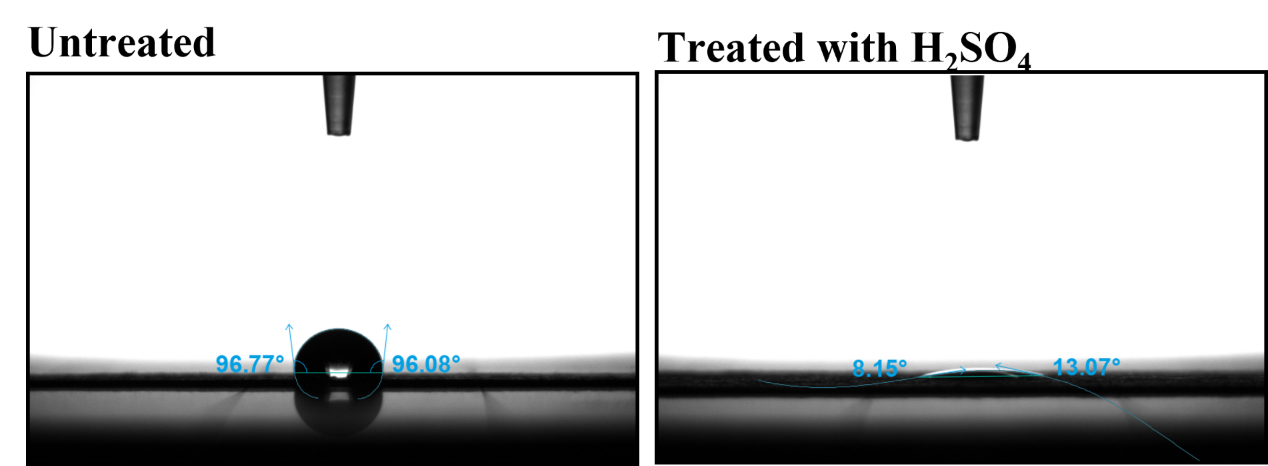


**Figure S17.** Hydrophily characterization of CP (left) and ECP (right).


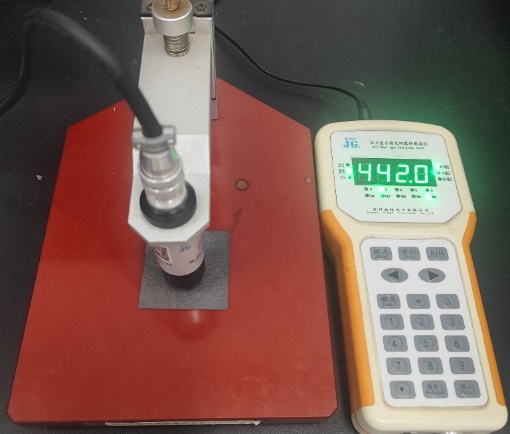


**Figure S18.** Sheet resistance (442.0 mΩ/sq) characterization of ECP.


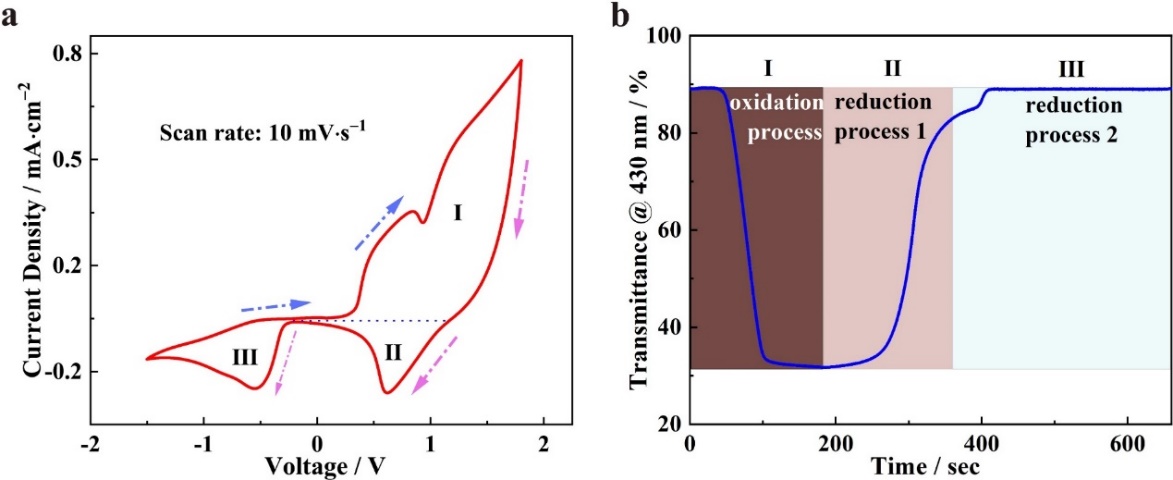


**Figure S19.** (a) CV curve of the Ni-DPNDI // ECP device at a scan rate of 10 mV·s^−1^ in the voltage of −1.5 V to 1.8 V. (b) The in-situ dynamic transmittance spectrum at 430 nm during the CV process.


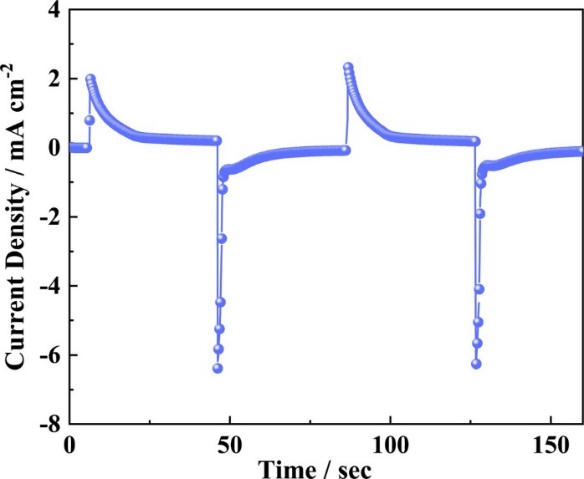


**Figure S20.** The diagram of current density change of Ni-DPNDI // ECP device by applying the square wave voltage of 1.5 and −1.5 V per 40 s.


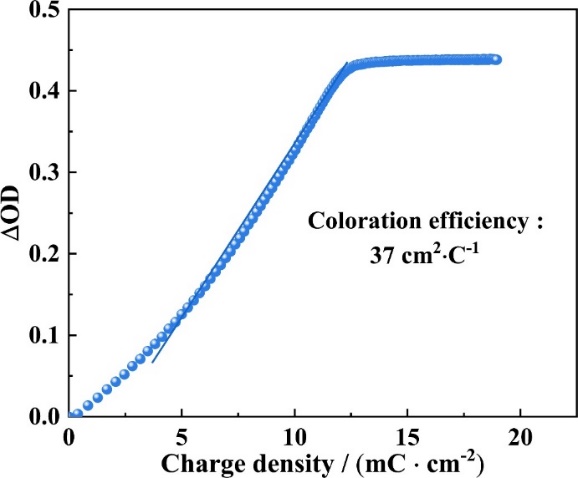


**Figure S21.** The relationship of ΔOD versus charge density. The inset is the coloration efficiency of the Ni-DPNDI // ECP device.


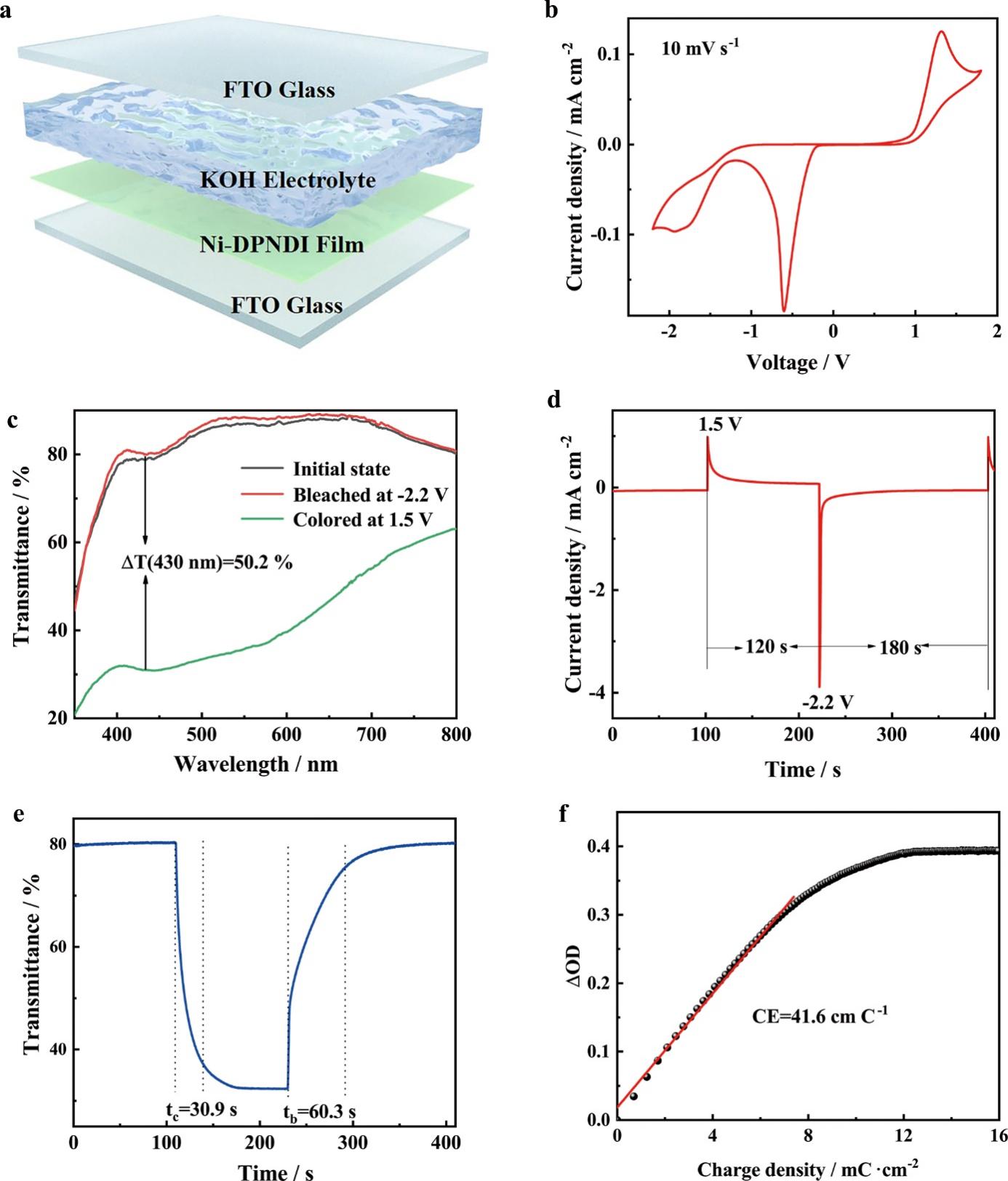


**Figure S22.** (a). Schematic diagram of Ni-DPNDI // FTO device with Ni-DPNDI electrode as the working layer, FTO as the counter electrode, and 0.1 M KOH solution as an electrolyte. (b). The CV curve of Ni-DPNDI // FTO device. (c). The UV-vis spectra of Ni-DPNDI // FTO device at its initial state, colored state (1.5 V), and bleached state (-2.2 V). (d). The diagram of current density change of Ni-DPNDI // FTO device by applying the square wave voltage of 1.5 and −2.2 V. (e). The in-situ transmittance change of the Ni-DPNDI // FTO device at 430 nm under the voltage at 1.5 V (colored state) or −2.2 V (bleached state). (f). The relationship of ΔOD versus charge density. The inset is the coloration efficiency of the Ni-DPNDI // FTO device.


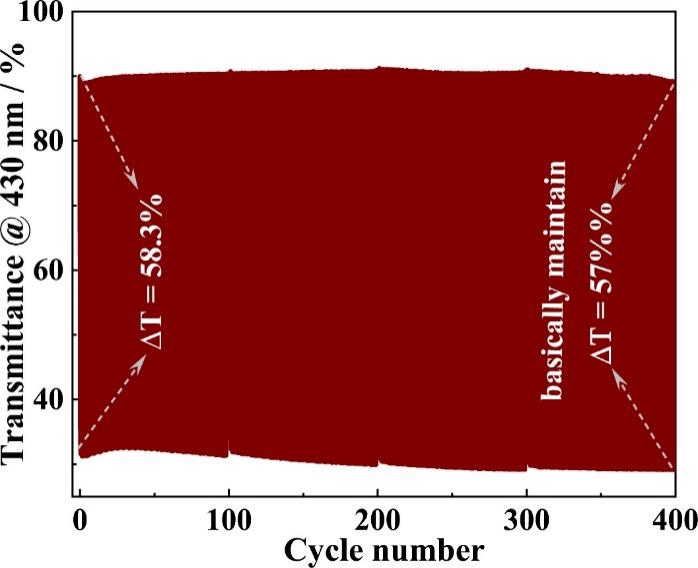


**Figure S23.** The in-situ transmittance change of the Ni-DPNDI // ECP device at 430 nm under the voltage at 1.5 V (colored state) or −1.5 V (bleached state) for 40 s in 0.1 M KOH electrolyte.


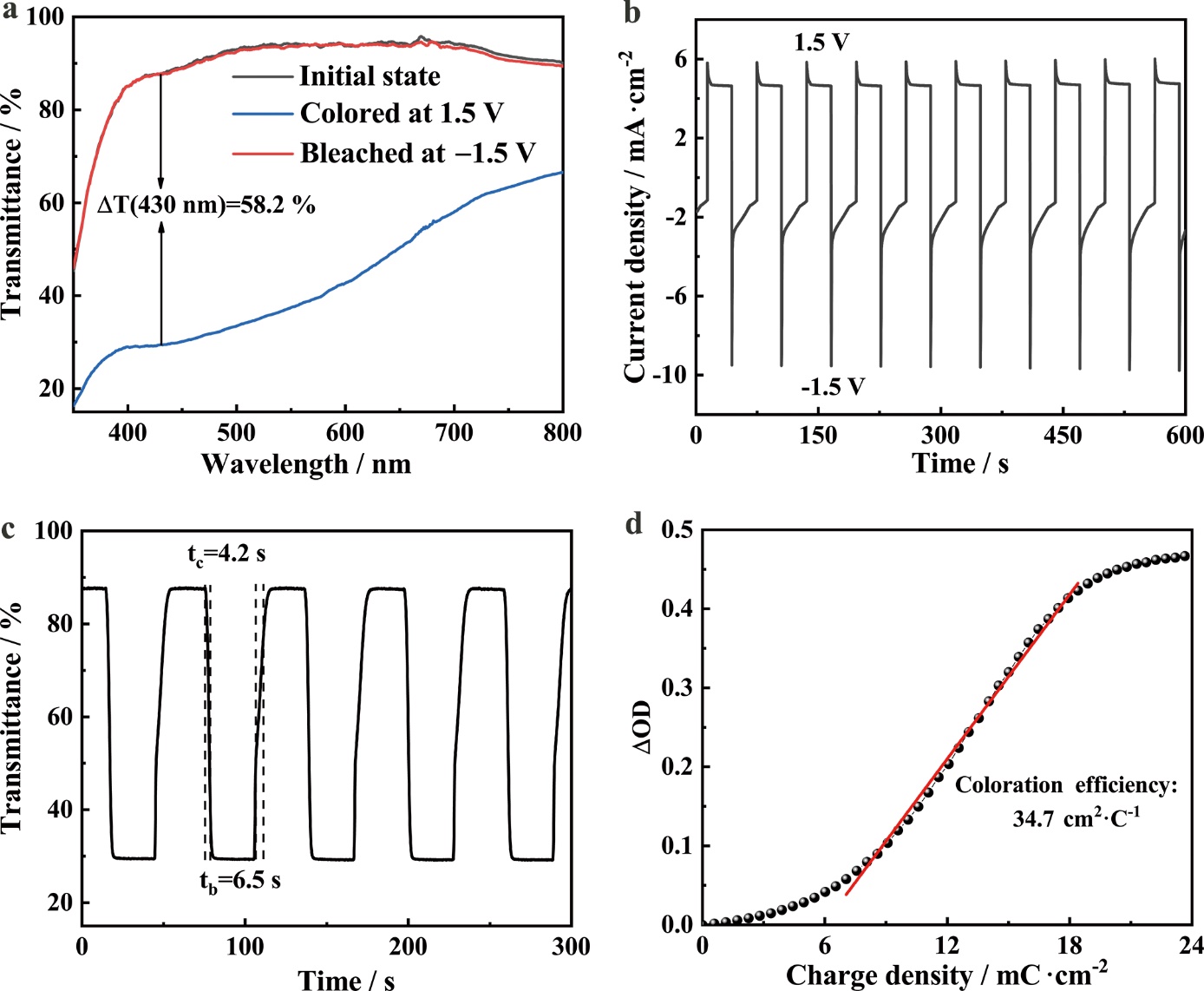


**Figure S24.** The EC performance of Ni-DPNDI // ECP device in 1 M KOH electrolyte. (a). The UV-vis spectra of Ni-DPNDI // ECP device. (b). The current density change of Ni-DPNDI // ECP device by switching the voltage cycle of 1.5 V and -1.5 V. (c). The transmittance change of Ni-DPNDI // ECP device at 430 nm by switching the voltage cycle of 1.5 V and -1.5 V. (d). The coloration efficiency of Ni-DPNDI // ECP device.


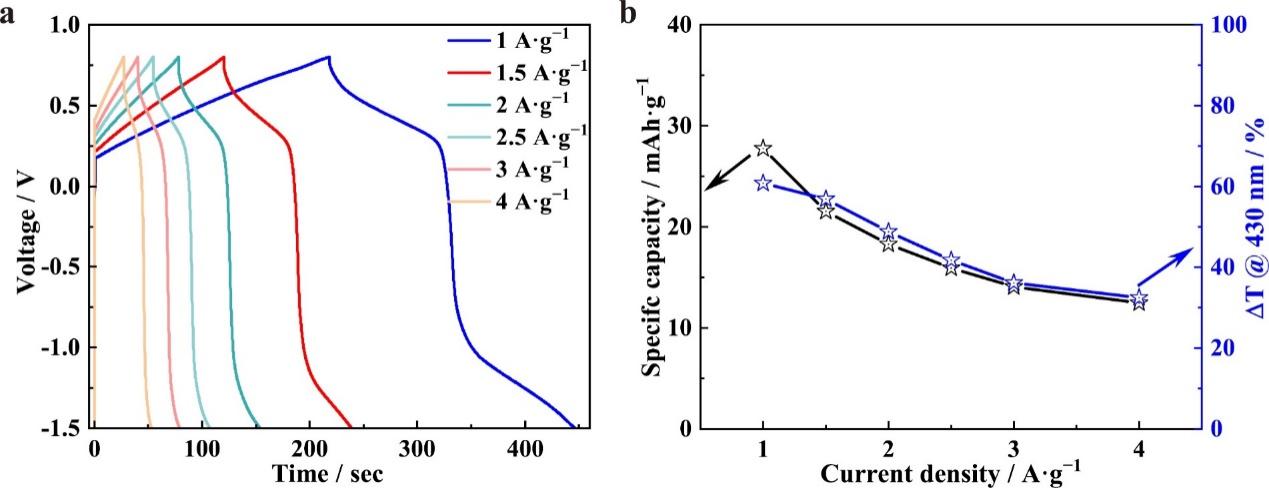


**Figure S25.** Energy storage performance of Ni-DPNDI // ECP device. (a) The galvanostatic charge-discharge curves at different current densities. (b) Optical modulation (blue) and corresponding capacitance (dark) of the Ni-DPNDI film at different charging currents.


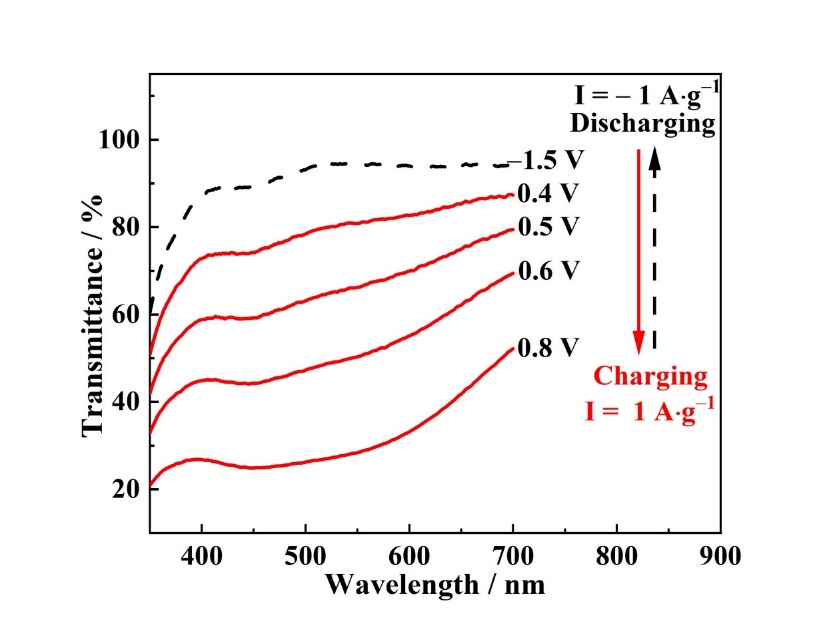


**Figure S26.** Transmittance spectra change of Ni-DPNDI // ECP device in the GCD process.

**
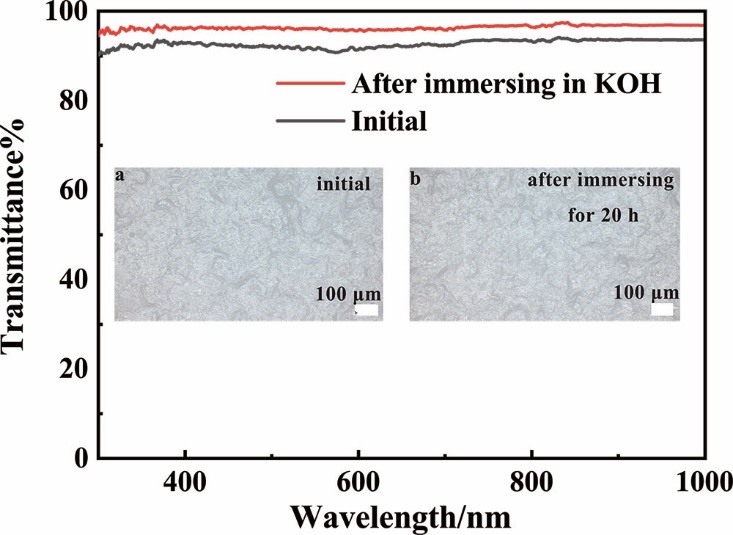
**

**Figure S27.** The UV-vis spectra of quartz glass before and after immersing in KOH electrolyte. The inset is optical photomicrographs of quartz glass.


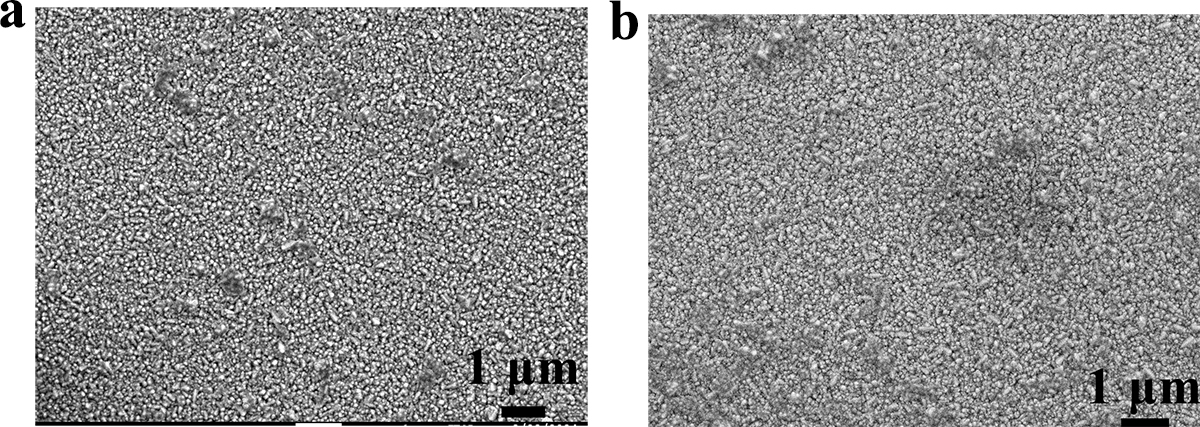


**Figure S28.** The SEM images of FTO glass before and after immersing in KOH electrolyte for 3 days.

The KOH can react with the SiO_2_ component of the glass to form a water-like glass of K_2_SiO_3_. However, in our alkaline electrolyte system, the amount of KOH (0.1 M) is lower, and it reacts slowly with SiO_2_ in glass. Moreover, the extent of reaction between KOH and glass is lower, which does not affect the surface and optical properties of glass. To prove the above result, we tested the photomicrographs and UV-vis spectra of the quartz glass and FTO glass before and after immersing it in the KOH electrolyte (Figures S25 and S26). The photomicrographs and UV-vis spectra of quartz glass illustrate that the surface and optical property of quartz glass have no obvious change after immersing it in the KOH electrolyte for 20 hours. Additionally, the SEM images (Figure S26) illustrate that the FTO layer is stable in the KOH alkaline electrolyte and the dense FTO layer has a good protective effect on its lower glass.

**Table S1.** Crystal data details for Ni-DPNDI MOF.

| Atom | x | y | z | Occupancy |
| --- | --- | --- | --- | --- |
| O1 | -1.12077 | 0.05471 | -0.05353 | 1 |
| O2 | -0.9474 | 0.05466 | -0.1212 | 1 |
| C3 | -1.4124 | 0.43498 | 0.48352 | 1 |
| C4 | -1.32538 | 0.53215 | 0.46692 | 1 |
| C5 | -1.41355 | 0.63339 | 0.48444 | 1 |
| O6 | -1.33706 | 0.66155 | 0.47236 | 1 |
| C7 | -1.52693 | 0.76432 | 0.58401 | 1 |
| C8 | -1.52655 | 0.82863 | 0.58258 | 1 |
| H9 | -1.25711 | 0.55616 | 0.454 | 1 |
| H10 | -1.54766 | 0.74015 | 0.65018 | 1 |
| H11 | -1.54761 | 0.85311 | 0.64825 | 1 |
| O12 | -1.44202 | 0.94595 | -0.38675 | 1 |
| O13 | -1.38627 | 0.94589 | -0.55714 | 1 |
| C14 | -1.98822 | 0.56503 | -0.08835 | 1 |
| C15 | -1.97627 | 0.46785 | -0.17612 | 1 |
| C16 | -1.98911 | 0.36659 | -0.08715 | 1 |
| O17 | -1.98117 | 0.33845 | -0.16419 | 1 |
| C18 | -2.08231 | 0.23562 | 0.03172 | 1 |
| C19 | -2.08089 | 0.1713 | 0.03126 | 1 |
| H20 | -1.96701 | 0.44384 | -0.24499 | 1 |
| H21 | -2.14719 | 0.25978 | 0.05622 | 1 |
| H22 | -2.14524 | 0.14683 | 0.05607 | 1 |
| H23 | -1.83612 | 0.05871 | -0.51043 | 1 |
| H24 | -0.48973 | 0.05877 | 0.16409 | 1 |
| H25 | -0.60265 | 0.10125 | 0.16992 | 1 |
| H26 | -1.17017 | 0.89875 | -0.60242 | 1 |
| C27 | -0.86354 | 0.93946 | -0.43386 | 0.5 |
| H28 | -1.05804 | 0.0714 | -0.55994 | 1 |
| H29 | -1.55982 | 0.92882 | 0.05799 | 1 |
| C30 | -1.43378 | 0.93949 | -0.13646 | 0.5 |
| H31 | -1.73322 | 0 | -0.16522 | 1 |
| H32 | -1.73186 | 0 | 0.37812 | 1 |
| H33 | -2.36307 | 0 | 0.01842 | 1 |
| H34 | -1.01836 | 0 | -0.36357 | 1 |
| C35 | -1.22992 | 0 | -0.22844 | 0.76 |
| C36 | -1.22834 | 0 | 0.23002 | 0.76 |
| C37 | -1.15027 | 0 | -0.10203 | 1 |
| C38 | -1.26744 | 0 | -0.1292 | 1 |
| C39 | -0.89851 | 0 | -0.1504 | 1 |
| C40 | -0.87092 | 0 | -0.26753 | 1 |
| H41 | -1.3781 | 0 | 0.26839 | 1 |
| H42 | -1.83473 | 0 | 0.26718 | 1 |
| H43 | -1.03551 | 0 | -0.23384 | 1 |
| H44 | -1.01836 | 0 | -0.36357 | 1 |
| H45 | -2.23318 | 0 | 0.03538 | 1 |
| H46 | -2.36307 | 0 | 0.01842 | 1 |
| C47 | -2.28967 | 0 | -0.01642 | 1 |
| C48 | -1.01637 | 0 | 0.29014 | 1 |
| C49 | -1.28892 | 0 | -0.30215 | 0.76 |
| C50 | -1.69787 | 0 | -0.28909 | 0.76 |
| C51 | -1.45755 | 0 | -0.3338 | 1 |
| C52 | -1.37655 | 0 | -0.26594 | 1 |
| C53 | -1.3334 | 0 | -0.54208 | 1 |
| C54 | -1.26583 | 0 | -0.62332 | 1 |
| C55 | -0.62344 | 0 | 0.1636 | 0.5 |
| C56 | -0.83647 | 0 | -0.37663 | 0.5 |
| C57 | -1.5 | 0.46737 | 0.5 | 1 |
| N58 | -1.5 | 0.66502 | 0.5 | 1 |
| C59 | -1.5 | 0.73158 | 0.5 | 1 |
| N60 | -1.5 | 0.86025 | 0.5 | 1 |
| C61 | -2 | 0.53263 | 0 | 1 |
| N62 | -2 | 0.33495 | 0 | 1 |
| C63 | -2 | 0.26838 | 0 | 1 |
| N64 | -2 | 0.13968 | 0 | 1 |
| Ni65 | -1 | 0.05297 | 0 | 1 |
| Ni66 | -1.5 | 0.94698 | -0.5 | 1 |

**Table S2.** The conductivity of Ni-DPNDI powder.

| Times | Temperature (K) | Conductivity (S·m^-1^) |
| --- | --- | --- |
| 1 | 300.0019 | 4.636992 |
| 2 | 300.0028 | 4.639748 |
| 3 | 299.9984 | 4.635079 |
| 4 | 300.0011 | 4.633732 |
| 5 | 300.0001 | 4.636746 |
| 6 | 300.0003 | 4.63296 |
| 7 | 300.0002 | 4.637187 |
| 8 | 300.0002 | 4.636735 |
| 9 | 300.001 | 4.635907 |
| 10 | 300.0001 | 4.638171 |
| 11 | 299.9978 | 4.63628 |
| 12 | 300.0009 | 4.635891 |
| 13 | 300.0017 | 4.637968 |
| 14 | 299.9988 | 4.634011 |
| 15 | 299.9985 | 4.632297 |
| 16 | 300.0019 | 4.634481 |
| 17 | 300.0013 | 4.633384 |
| 18 | 299.9993 | 4.634281 |
| 19 | 299.9992 | 4.634549 |
| 20 | 299.9986 | 4.635296 |
| Mean value | - | 4.635584 |

**Table S3.** Comparison of electrochromic performance of EC MOF films.

| MOFs  film | Color change | ΔT / λ | t_c_ / t_b_ s | CE / (cm^2^ / C) | Cycling number of the film (Retains %) | Electrolyte | Extra functionality | reference |
| --- | --- | --- | --- | --- | --- | --- | --- | --- |
| UiO-67-dumpy film | white  →  yellow | 57.3%  /  430 nm | 16.7 / 8.0 | 89 | 10 cycles | KCl/H_2_O | sensor | 1 |
| NU-901 MOF film | yellow  →  Blue | 62%  /  587 nm | 12 / 5 | 204 | 60 (38%) | 0.1 M TBA PF_6_  /  CH_2_CL_2_ solution | NA | 2 |
| Zn(NDI-X) film | blue region  visible region | NA/  470-500 nm  NA/  600-700 nm. | NA | 100  60-90 | 50 | 0.1 M [(nBu)_4_N]PF_6_ / DMF | NA | 4 |
| Ni-NDISA film | transparent →  dark | NA | NA | NA | NA | 0.1 M [(nBu)_4_N]PF_6_ / DMF | NA | 5 |
| Zn-NDI-74 film | canary yellow  →  dark brown | 21% / 475 nm  32% / 609 nm | 14 / 23  3 / 91 | 117  52 | NA | 0.1 M [(nBu)_4_N]PF_6_ / DMF | NA | 9 |
| Cu-TCA film | light yellow →  dark blue | 65%  /  700nm | 4.8 / 3.3 | NA | 1000 (95%) | 0.1 M LiClO_4_ / PC | NA | 10 |
| Ni-MOF-125 film | light yellow →  reddish brown | 44.4%  /  550 nm | 24.5 / 23.5 | NA | 650 | 1 M LiClO_4_ / PC | NA | 12 |
| PrGO / HKUST-1  film | dark blue  →  pale blue | 38.5%  /  500 nm | 1.3 / 1.4 | 176.8 | 900 (83.4%) | 1 mM LiClO_4_  /  ACN | NA | 13 |
| HKUST-1  film | bright blue →  light blue | 24.7%  /  500 nm | 23 / 31.9 | 80.6 | NA |  |  |  |
| HKUST-1 film | bright blue →  light blue | 3.5%  /  633 nm | 56.5 / 25.0 | 36.7 | NA | 0.5 M LiClO_4_  /  PC | NA | 14 |
| HKUST-1  /WO_3_ film | colorless  →  blue | 76.6%  /  633 nm | 8.3 / 2.2 | 296.4 | 1000 (85.9%) |  |  |  |

| Zn-PMDI film | colorless  → blue → pink | 89.5%  /  714 nm | NA | 753 | NA | 0.5 M  KPF_6_ / DMF | NA | 15 |
| --- | --- | --- | --- | --- | --- | --- | --- | --- |
| Zn-NDI  film | pale yellow → orange → gray-blue | 97.1%  /  472 nm | 3.7 / 4 | 610 | 50 (96%) |  |  |  |
| Zn-PDI film | red →  light green  → blue | 96.4%  /  746 nm | 1.6 / 2.6 | 941 ± 35 | 150 (98%) |  |  |  |
| Zn_2_(PDICl_4_)_2_ film | orange  →  dark blue | 0.085  /  645 nm | < 14 | 104.8 | 100 cycles | LiTFSI  ‑  EMITFSI | NA | 11 |
|  | orange  →  cyan | 0.352  /  650 nm | NA | NA | NA | EMITFSI | NA |  |
| Ni-CHNDI film | transparent → red → dark blue | 73%  /  720 nm | 2.1 / 1.9 | 260.3 | 500 (91%) | 0.1 M NaClO_4_  /  PC solution | NA | 3 |
| HKUST-1  film | light blue  →  bright blue | 64%  /  460 nm | 8 / 9 | NA | 100 (99.5%) | 0.3 M LiTFSI  /  EMITFSI | NA | 6 |
| Zn MOF-74 film | brown  →  yellow | 31%  /  600 nm | 10 / 7 | NA | 50 |  |  |  |
| Ni_3_(HITP)_2_ film | dark blue  →  light yellow | 38.8%  /  524 nm | 9.4 / 6.8 | 100 | 100 (95%) | 1 M LiClO_4_/PC | NA | 8 |
| Mg-PDI MOF film | red  →  Purplish-red →  Purple | 42%  /  720 nm | 9.2 / 0.6 | 305 | 5000 (93.4%) | ionic liquid | wide temperature range | 7 |
| Ni-IRMOF-74@MBA film | transparent  →green→  dark-brown | 58%  /  900 nm | 1.9 / 2.0 | 331.0 | 4500 (95.7%) | 1 M LiClO_4_/PC | NA | 17 |
| Ni-BPY film | transparent  →  brown-dark | 70%  /  430 nm | 9.6 / 6 | 32 | 500 (90%) | 1 M KOH | patterned display | 16 |
| Ni-DPNDI film | transparent  →  dark-brown | 60.8%  /  430 nm | 7.9 / 6.4 | 28.9 | 2000 (100%) | 0.1 M KOH | energy storage | This work |

**Table S4.** Comparison of electrochromic performance of MOF-based EC devices.

| MOFs EC Device  WE // CE | Color change | EC performance | Cycling number of device (Retains %) | reference |
| --- | --- | --- | --- | --- |
| Zn_2_(PDICl_4_)_2_  //  ITO | orange  →  dark blue | NA | NA | 11 |
|  | orange  →  cyan |  |  |  |
| Ni-CHNDI film  //  FTO | Transparent  → red →  dark blue | NA | NA | 3 |
| 2×5 cm^2^  HKUST-1 film  //  Zn MOF-74 film | light blue  →  bright blue | NA | NA | 6 |
|  | brown  →  yellow |  |  |  |
| 2.5×2.5 cm^2^  Ni_3_(HITP)_2_ film  //  FTO | dark blue  →  light yellow | NA | NA | 8 |
| 6×8 cm^2^  Mg-PDI  //  ITO | red  → Purplish-red →  Purple | ΔT = 28%, t_c_ = 4.2 s,  t_b_ = 5 s  CE = 331.08 cm^2^ / C | 200 (95.8%) | 7 |
| 5 × 3 cm^2^  Ni-IRMOF-74@MBA  //  FTO | transparent  →green→  dark-brown | ΔT430nm = 61%  t_c_ = 2.3 s  t_b_ = 7.9 s  CE = 37 cm^2^ / C | 1200 (85%) | 17 |
| 5 × 3 cm^2^  Ni-BPY  //  FTO | transparent  →brown-dark | ΔT_430nm_ = 60% | NA | 16 |
| **10×10 cm^2^**  **Ni-DPNDI**  **//**  **ECP** | transparent  →  dark-brown | ΔT_430nm_ = 59%  t_c_ = 8.2 s  t_b_ = 10.9 s  CE = 37 cm^2^ / C | 400 (98%) | This work |

**Table S5.** Comparison of EC performance of Ni-DPNDI film and reported NiO materials.

| NiO-based EC film sample  (method) | ΔT / λ | t_b_/s | t_c_/s | CE /  (cm^2^ / C) | Cycling  Numbers (Retains %) | reference |
| --- | --- | --- | --- | --- | --- | --- |
| NiO nanoparticles film  (Solvothermal ) | 63.6 /  550 nm | 11.5 | 9.5 | 42.8 | 1000 (90.8% )  5000 (56.4% ) | *Nano Energy* **2015**, *12*, 258−267. |
| Ni−V Oxides film  (Chemical bath deposition) | 36 /  630 nm | 38.87 | 9.03 | 16.95 | 2000 (91.95%) | *ACS Appl. Mater. Interfaces* **2021**, *13*, 57403−57410. |
| Nanostructured NiO film  (Hydrothermal) | 67 /  550 nm | 5.2 | 6.6 | 92.0 | 600 (40% ) | *J. Mater. Chem. A* **2013**, *1*, 4286−4292. |
| NiO nanoflake film  (Hydrothermal) | 40 /  632.8 nm | 1.8 | 2.7 | 63.2 | NA | *J. Mater. Chem. A* **2015**, *3*, 20614−20618. |
| Layer-stacked NiO  nanowire/sheet film  (Hydrothermal) | 93.4 /  550 nm | 9.5 | 12.2 | 72.1 | 1000 (91.2% ) | *Nanoscale*, **2023**, *15*, 8685−8692. |
| NiO/Ni(OH)_2_-based film  (Electroless nickelde position) | 64.3 /  550 nm | 14.1 | 3.1 | 12.0 | NA  (CV:420 cycles) | *ACS Omega* **2022**, *7*, 43, 39090−39096. |
| Cu-doped NiO film  (Electrode position) | 57.1 /  550 nm | 1.8 | 2.3 | 13.8 | NA  (CV:100 cycles) | *Electrochim. Acta* **2019**, *295*, 645−654. |
| N-doped NiO film  (Magnetron sputtering) | NA | 14.5 | 18.7 | 33.0 | NA | *Thin Solid Films* **2013**, *527*, 26−30. |
| Ni hydroxide film (Galvanostatic electrochemical reduction) | 83.2 /  432 nm | 9.5 | 12 | 29.0 | NA  (CV:50 cycles) | *J. Solid State Electrochem.* **2014**, *18*, 3359−3367. |
| NiO film  (Electrodeposition) | NA | 2.0 | 1.7 | 107 | NA (CV:10000 cycles Tc/b ) | *J. Alloys Compd.* **2010**, *489*, 667−673. |
| NiO film  (E-beam evaporation) | 38.8 /  550 nm | NA | NA | 24.8 | NA (CV:100 cycles) | *Crystals* **2021**, *11(6)*, 615 |
| Zn-doped NiO film  (Sol-gelspincoating) | 59.8 /  500 nm | 3.0 | 8.0 | 33.6 | NA (CA:  20000 s) | *Electrochem. Sci.* **2020**, *15*, 4065−4071. |
| NiO nanorods film  (Vapor deposition technique) | 60.0 /  630 nm | 1.2 | 1.3 | 43.3 | 1050 (78.3%) | *Sol. Energy Mater. Sol. Cells* **2013**, *112*, 91-96. |
| Porous NiO  (Sol-gel) | 51.0 /  500 nm | 5 | 7 | 40 | 3000 (90%) | *J. Mater. Chem. C* **2018**, *6*, 4952−4958. |
| NiO@C film  (Pyrolysis and spray coating) | 39.2 /  525 nm | 3.79 | 1.6 | 108.3 | 1500 (85.5% ) | *ChemElectroChem* **2022**, *9*, 202200001. |
| NiO@C film  (Hydrothermal and pyrolysis) | 35 /  550 nm | 4.5 | 8.5 | 113.5 | 20000 (90.1% ) | *ACS Sustainable Chem. Eng.* **2020**, *8*, 12222−12229. |
| N-C@NiO film  (Hydrothermal and pyrolysis) | 54.5 /  550 nm | 7.1 | 6.5 | 60.5 | 1000 ( 72% ) | *ACS Appl. Mater. Interfaces* **2021**, *13*, 4133−4145. |
| NiO@C film  (Hydrothermal and pyrolysis ) | 60.6 /  550 nm | 0.25 | 0.46 | 113.5 | 20000 (90.1% ) | *Mater. Horiz.* **2019**, *6*, 571−579. |
| Cubic NiO Film  (ESD) | 83.2 /  550 nm | NA | NA | 61.5 | NA | *J. Phys. Chem. Lett.* **2023**, *14*, 2284−2291. |
| **Ni-DPNDI**  **(ESD)** | 60.8 /  430 nm | 6.4 | 7.9 | 28.9 | 2000 (100%) | This work |

**Table S6.** Comparison of EC performance of Ni-DPNDI//ECP and reported NiO-based EC devices.

| EC Device  WE // CE | ΔT /% | t_b_ / s | t_c_ / s | CE /  (cm^2^ / C) | Cycling  numbers | Reference |
| --- | --- | --- | --- | --- | --- | --- |
| NiO // FTO | 47 | 6.7 | 2.7 | 85.3 | NA | *J. Solid State Electrochem.* **2021**, *25*, 821 |
| NiO // TiO_2_ | 74 | NA | NA | 61.5 | NA | *J. Phys. Chem. Lett.*,  **2023**, *14*, 2284 |
| NiO // TiO_2_ | 80.3 | 34.8 | 34.2 | 31.7 | NA | *Nanoscale*, **2023**, *15*, 8685-8692 |
| WO_3_ // NiO | 75 | 10 | 13.1 | 131.9 | 100 | *Nanoscale*, **2016**, *8*, 348 |
| WO_3_ // NiO | 46 | 3.1 | 4.6 | 90 | 2500 | *Sci. Rep.*, **2020**, *10*, 8430 |
| NiO // MnO_2_ | 57 | 12 | 12.1 | 28.5 | 200 | *J. Mater. Chem. A*,  **2021**, *9*, 6451 |
| AgNWs // NiO | 14.5 | NA | NA | 51.9 | 2000 | *Adv. Energy Mater.*,  **2018**, *8*, 1800069 |
| NiO // Li_4_Ti_5_O_12_ | 55 | 7.4 | 6.5 | NA | 600 | *J. Mater. Chem. A*,  **2021**, *9*, 6451 |
| **Ni-DPNDI // ECP** | 59 | 10.9 | 8.2 | NA | 400 | This work |

References

[1] S. Huang, Y. Liu, P. Huang, F. Wu, L. Mao, *Chem. Eur. J.* **2023**, *29*, e202300263.

[2] C.Kung, T. C. Wang, J. E. Mondloch, D. Fairen-Jimenez, D. M. Gardner, W. Bury, J. M. Klingsporn, J. C. Barnes, R. Van Duyne, J. F. Stoddart, *Chem. Mater.* **2013**, *25*, 5012.

[3] R. Li, K. Li, G. Wang, L. Li, Q. Zhang, J. Yan, Y. Chen, Q. Zhang, C. Hou, Y. Li, *ACS Nano* **2018**, *12*, 3759.

[4] C. R. Wade, M. Li, M. Dincă, *Angew. Chem. Int. Ed.* **2013**, *52*, 13377.

[5] K. AlKaabi, C. R. Wade, M. Dincă, *Chem* **2016**, *1*, 264.

[6] I. MjejriP, C. M. Doherty, M. Rubio-Martinez, G. L. Drisko, A. Rougier, *ACS Appl. Mater. Interfaces* **2017**, *9*, 39930.

[7] Z. Lu, R. Li, L. Ping, Z. Bai, K. Li, Q. Zhang, C. Hou, Y. Li, W. Jin, X. Ling, H. Wang, *Cell Rep Phys Sci.* **2022**, *3*, 100866.

[8] L. Pan, R. Li, C. Zhang, Z. Lu, K. Li, Q. Zhang, C. Hou, Y. Li, H. Wang, *ACS Appl. Electron. Mater.* **2022**, *4*, 2915.

[9] X. Wu, K. Wang, J. Lin, D. Yan, Z. Guo, H. Zhan, *J. Colloid Interf. Sci.* **2021**, *594*, 73.

[10] J. Liu, X. Y. D. Ma, Z. Wang, L. Xu, T. Xu, C. He, F. Wang, X. Lu, *ACS Appl. Mater. Interfaces* **2020**, *12*, 7442.

[11] A. Mazel, L. Rocco, N. Penin, A. Rougier, *Adv. Optical Mater.* **2023**, *11*, 2202939.

[12] N. Zhang, Y. Jin, Q. Zhang, J. Liu, Y. Zhang, H. Wang, *Ionics* **2021**, *27*, 3655.

[13] D. Mohanadas, T. B. S. A. Ravoof, Y. Sulaiman, *Sol. Energ. Mat. Sol. C.* **2020**, *214*, 110596.

[14] D. Mohanadas, N. I. A. Zainudin, Y. Sulaiman, *Chem. Eng. J.* **2022**, *428*, 130989.

[15] A. Kumar, J. Li, A. K. Inge, S. Ott, *ACS Nano* **2023**, *17*, 21595.

[16] J. Feng, X. Wang, Y. Luo, J. H. Wang, Z. P. Wang, C. Y. Wei,; G. Cai, *ACS Appl. Mater. Interfaces* **2024**, *16*, 1170.

[17] X. Fan, S. Wang, M. Pan, H. Pang, H. Xu, *ACS Energy Lett.* **2024**, *9*, 2840.
